# Sorp‐Vection‐Based Membrane Silicone Oil Purification

**DOI:** 10.1002/anie.202516848

**Published:** 2025-11-12

**Authors:** Jinyoung Kim, Yuhe Cao, Wulin Qiu, Zhongyun Liu, Steven Schlosser, Reza Haghpanah, Dimitris Katsoulis, Jay Rose, Seo‐Yul Kim, Hammed A. Balogun, Ryan Lively, William J. Koros

**Affiliations:** ^1^ School of Chemical & Biomolecular Engineering Georgia Institute of Technology 311 Ferst Drive Atlanta GA 30332 USA; ^2^ Dow Chemical Company 2211 HH Dow Way Midland MI USA

**Keywords:** Membrane separation, Silicone oil purification, Siloxane, Sorp‐vection

## Abstract

“Sorp‐vection” is a membrane separation technique that synergistically combines sorption with convective flow mechanisms. Beyond its conceptual discussion, we demonstrate a sorp‐vection separation achieved in a gas–liquid system, where permeation of a gas directly drives selective permeation of an organic solute across a dense polymer layer overcoming osmotic limitations of conventional membrane processes. Here, a long‐standing challenge in silicone oil production is addressed, in which residual cyclic oligosiloxanes are removed from silicone oil streams through permeation of CO_2_ across an optimally crosslinked PDMS selective layer. A lab‐scale 1^st^ generation Sorp‐vection system demonstrated, with a separation factor above 15 to remove D4 (octamethylcyclotetrasiloxane) from low‐concentration feeds using both lab‐grade silicone oil (Sigma‐Aldrich) and an industrial‐grade feed (DOW‐SFD). Good agreement was found with a predictive model based on liquid D4 and high‐molecular‐weight silicone oil sorption data in crosslinked PDMS. This proof‐of‐concept study introduces the sorp‐vection strategy, expanding it from conventional two‐component systems to a three‐component configuration in which convective flow is introduced as an independent driving entity. Addressing concentration polarization in next‐generation versions of the sorp‐vection process is expected to ensure stable long‐term performance and to establish sorp‐vection as a transformative approach for industrial purification.

## Graphical abstract

This work presents the first Sorp‐vection‐based membrane separation for silicone oil purification, coupling sorption‐driven selectivity with convective transport. Our strategy enables highly selective removal of residual cyclic siloxanes, particularly D4, overcoming the inherent trade‐offs and limitations associated with conventional membrane processes in the silicone industry.

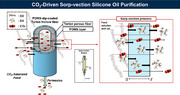

## Introduction

Separation technologies have a significant impact on chemical process economics,^[^ ^1^, ^2^, ^3^, ^4^ ^]^ and membrane separations can reduce these costs via energy efficiency, give a high separation selectivity and reductions in system footprint.^[^ ^5^, ^6^ ^]^ These advantages arise from their molecular‐level selectivity, as separations are governed by penetrant size and solubility differences.^[^ ^7^, ^8^, ^9^ ^]^ Despite these strengths, many challenging separations remain, particularly when overcoming separation inefficiencies in dilute feed systems, especially liquid phase feeds. Our current study reports a way to overcome this challenge using the essential cyclic siloxane contaminant removal from silicone oils as a key example.^[^ ^10^, ^11^ ^]^ In such systems, crosslinked polydimethylsiloxanes (PDMS) are often considered as practical and scalable membrane matrices; however, high osmotic pressure differences between feed and product streams exist in these cases, and this osmotic barrier to meaningful fluxes is further compounded by the low diffusivity of cyclics in PDMS.^[^ ^12^, ^13^, ^14^, ^15^ ^]^ Here, we demonstrate an unusual pressure‐driven process, sorp‐vection,^[^ ^16^ ^]^ combines sorption into the selective membrane matrix with convective flow in a single mechanism to enhance efficiency and selectivity beyond conventional membrane processes using a selected “Sorp‐vection agent” illustrated by CO_2_, which has high permeability in the membrane matrix. This proof‐of‐concept establishes sorp‐vection as a viable framework for gas‐assisted liquid separations, paving the way for future industrial realization as a broadly applied tool.

Polysiloxanes have valuable properties in many industrial and consumer applications^[^ ^17^, ^18^, ^19^, ^20^, ^21^ ^]^; however, long‐standing challenges exist to economic removal of persistent cyclic oligo‐siloxanes (referred to as “cylics” in this work). Studies show siloxane contaminants (e.g., octamethylcyclotetrasiloxane (D4), decamethylcyclopenta‐siloxane (D5), dodecamethylcyclo‐hexasiloxane (D6), shown in Scheme S1) exhibit attractive material properties that make them useful in numerous consumer applications.^[^ ^22^, ^23^ ^]^ They are also crucial as precursors for the synthesis of silicone polymers.^[^ ^24^ ^]^ These benefits should be balanced with their potential environmental contamination and bioaccumulation.^[^ ^25^, ^26^, ^27^ ^]^ Different regions of the world have imposed regulations on the levels of cyclic siloxanes in consumer products^[^ ^24^, ^28^ ^]^ and thus the silicone industry has been adopting in producing “low cyclics” products. These cyclics form from thermodynamically favorable backbiting reactions during silicone oil polymerization and are typically at low concentrations (≤10 wt%) within the final silicone products, requiring costly post‐synthesis removal processes.^[^ ^29^, ^30^, ^31^ ^]^ Approaches to address these challenges include: 1) catalytic control^[^ ^32^ ^]^; 2) Solvent‐assisted stripping^[^ ^31^ ^]^; 3) Deep eutectic solvent (DES) absorption technique^[^ ^33^ ^]^; and, 4) Reverse osmosis (used in cyclic removal in rich feeds of 90% D4 in silicone oil).^[^ ^34^ ^]^ Despite these advances, current approaches still fall short of meeting industrial demands, and a scalable solution for dilute cyclics removal that can be integrated into existing silicone oil polymerization processes is needed. Here, we achieved enhanced separation performance between cyclics and silicone oil, even with low‐concentration feeds; However, we also identified a key hurdle and described our strategy to address it.

The discovery of sorp‐vection and subsequent studies of it focused on two‐component systems, comprising a feed solvent and a species to be separated. Our current study expands the approach to a three‐component sorp‐vection configuration in which convective flow is not inherently part of the feed permeation but is introduced as an independent, motivating entity. This advancement allows for deeper insight into the role of convective flow in sorp‐vection theory and how it can create its own challenge if not dealt with properly. Our study, therefore, strengthens the theoretical foundation and practical applicability of sorp‐vection for complex feed systems as a transformative approach, not only for cyclics removal in silicone oil production but also with potential for other complex separation systems.^[^ ^9^, ^35^, ^36^, ^37^ ^]^

## Theory

### Molecular Transport via Sorp‐Vection

**Figure 1:**
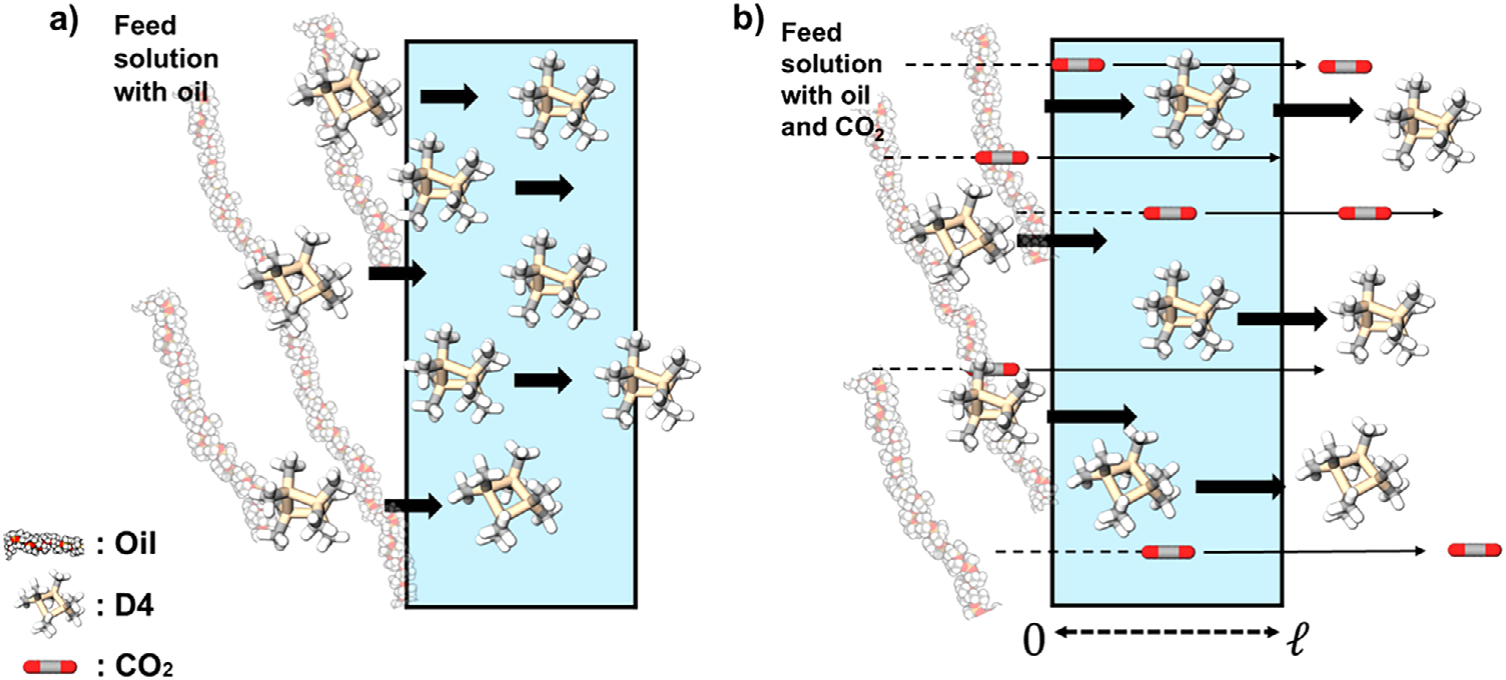
Illustrative sorp‐vection model schematic: a) The crosslinked membrane preferentially sorbs the target organic solute (D4) from the feed due to its high partition coefficient, compared to the high molecular weight oil. Moreover, the high molecular weight silicone oil, with limited solubility within the membrane, is not convected to the permeate. b) The convective flux of the sorp‐vection agent (CO_2_) causes transport of the sorbed D4 toward the downstream side, thereby enhancing its concentration relative to the upstream. Light blue area represents PDMS selective layer.

The sorp‐vection model describes flux of the sorp‐vection agent (CO_2_) by Fick's law when added to initial feed solutions comprising oil and impurity, Octamethylcyclotetrasiloxane (D4). Both of these components are large and have low diffusion coefficients.^[^ ^34^ ^]^ Fortunately, CO_2_, the sorp‐vection agent, can be engineered to convectively transport the sorbed D4 impurity from the feed to the permeate side of the membrane. On the other hand, the high molecular weight silicone oil, which exhibits low solubility and mobility within the membrane matrix, is largely excluded, enabling selective D4 removal with the CO_2_ permeant (Figure 1).

To develop our sorp‐vection theory, we begin with Fick's law for component i, which describes the permeant i flux through a membrane with respect to a fixed frame of reference as equal to the sum of bulk and diffusional fluxes. This is the same framework discussed in *Transport Phenomena by Bird, Stewart, and Lightfoot* for a binary case.^[^ ^38^ ^]^ We have simply generalized this framework to include the case of the ternary (D4 = A, CO_2 _= B, oil = C, and membrane = P) for transport relative to a fixed frame of reference. The membrane is not moving at steady state, but the other components i = *A*; *B*; *C* have flux components contributed by the sum of diffusive and bulk flow contributions. This relationship is expressed as follows:

(1) $$n_{i}=j_{i_{Diff}}+n_{i_{Bulk}}$$

(2) $$j_{i_{Diff}}=-\rho D_{i,m}\;\left(d\omega_{i,m}/\mathrm{dz}\right)$$

here, *n_i_* is the total flux of component *i*; $j_{i_{Diff}}$ is the diffusion flux of component *i*; *D*_i,*m*_ is the effective Fickian diffusivity of component *i* in the membrane; ω_i,*m*_ is the mass fraction of component *i* in the membrane; *z* is the transport direction coordinate.

The diffusive fluxes of both D4 and high molecular weight silicone oil are very low, due to their low diffusion coefficients,^[^ ^34^ ^]^ and flux of the membrane material itself is zero at a steady state (*n_P_* =  0). On the other hand, the diffusive flux of CO_2_ is high due to significant sorption and a high diffusion coefficient due to its high critical temperature (*T*_c_) and compact size, respectively.

When one of the components (i.e., CO_2_ in this case) has such a high permeation flux compared to the others, sorption‐vection can occur due to the appearance of a tailored bulk flow effect in this paper.^[^ ^39^, ^40^ ^]^ In general, when bulk flow effects are considered, the following equations for the flux of components *A*, *B*, and *C* apply.

(3) $$n_{A}=-\rho D_{A,m}\frac{d\omega_{A,m}}{dz}+\omega_{A,m}\left(n_{A}+n_{B}+n_{C}\right)$$

(4) $$n_{B}=-\rho D_{B,m}\frac{d\omega_{B,m}}{dz}+\omega_{B,m}\left(n_{A}+n_{B}+n_{C}\right)$$

(5) $$n_{C}=-\rho D_{C,m}\frac{d\omega_{C,m}}{dz}+\omega_{C,m}\left(n_{A}+n_{B}+n_{C}\right)$$

The flux of the sorp‐vection agent (component B, CO_2_), is primarily due to $j_{CO2_{Diff}}$. In this case $n_{B}=j_{CO2_{Diff}}+n_{CO2_{bulk}}$. This approximation allows using pure component CO_2_ permeation data with pure component CO_2_ sorption data to estimate n_CO2_ in Equation (1) and greatly simplify the sorp‐vection analysis. Moreover, we found this flux to be reasonably approximated by the pure component value in an optimally crosslinked membrane. In this case, Equations (3)–(5) can be simplified taking *n_B_* to comprise the total bulk flow term. Diffusion terms $j_{A_{Diff}}$ and $j_{C_{Diff}}$ can be neglected due to the low diffusivity and likely low diffusive fluxes. As will be shown later, this is consistent with the experimental value of $n_{B}\approx 53.3\frac{kg}{m^{2}hr}$, which is 100 times bigger than the sum of $n_{A_{bulk}}+n_{C_{bulk}}$, allowing *n_A_* and *n_C_* in Equations (3)–(5) to be neglected, so the data are based on experimental values, and will be discussed later in Equation (10). The summarized sorp‐vection equations can, therefore, be reorganized as follows:

(6) $$n_{A}\approx \omega_{A,m}\left[n_{B}\right]$$

(7) $$n_{B}\approx -\frac{\;\rho D_{B,m}}{1-\omega_{B,m}}\frac{\partial \omega_{B,m}}{\partial x}\approx \;\frac{P_{B}\left[\Delta p_{B}\right]}{\left(1-\omega_{B,m}\;\right)\;\ell }$$

(8) $$n_{C}\approx \omega_{C,m}\left[n_{B}\right]$$

Here, where $P_{B}$ is the CO_2_ permeability through the membrane, ℓ is the membrane thickness, ρ is the density and Δ*p_B_* is the transmembrane CO_2_ partial pressure. The separation factor on a CO_2_‐free basis (which is ultimately the separation factor of interest), can be calculated using the CO_2_‐free mass fraction of components at the permeate and feed sides of the membrane and be expressed as:

(9) $$S.F.=\frac{{\left(n_{A}/n_{C}\right)}_{Permeate}}{{\left(\omega_{A}/\omega_{C}\right)}_{Feed}}$$

Although highly convenient for estimation purposes, the full Equations (3)–(5) can also be used for more accurate calculations without excessive difficulty (see Equations (S3)–(S5)). As will be discussed later, the estimated CO_2_ flux using the permeability with the pure CO_2_ present may be too simplified if the viscous oil that is being carried through the selective layer encounters difficulty in flowing from the downstream face of that layer *through the tight pores of the porous support*. In this case, additional mass transfer resistance is created due to accumulation of viscous, almost immobile oil in the porous support, slowing down the flux of the CO_2_ and the D4 from the downstream face of the selective layer of the PDMS. We will show evidence that this situation is occurring and provide a solution to the problem.

## Results and Discussion

### Formation of Optimally Crosslinked Membrane of PDMS on Torlon Hollow Fiber

Torlon (polyamide‐imide (PAI)) asymmetric hollow fibers serve as the supporting substrate for the dual‐layer separating fiber in the sorp‐vection process. The outer PDMS layer on the hollow fiber membranes acts as the selective entity that enables preferential permeation of cyclic siloxanes, component A, relative to the high molecular weight oil, component C. This means that the separation performance is critically dependent on optimally crosslinked membrane. To establish the optimal crosslinking conditions, we conducted systematic experiments varying the molecular weight, solution concentration, and dipping time of the PDMS layer. The detailed coating procedure and rationale for solvent selection are provided in the Supporting Information.

**Figure 2:**
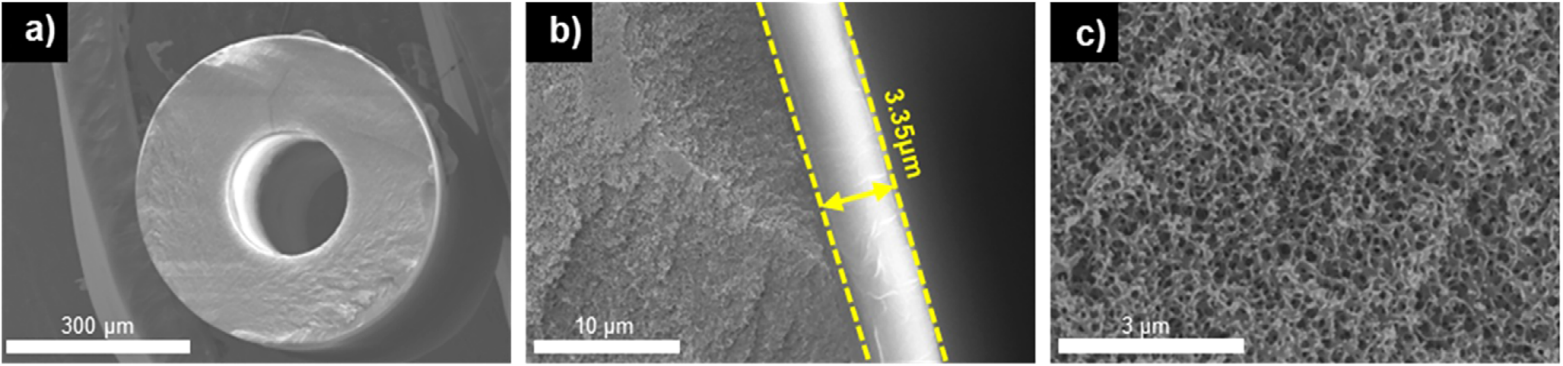
SEM analysis on PDMS‐coated monolith hollow fiber, a) fiber cross‐section; b) fiber wall with PDMS layer; c) Torlon porous support layer with uniform small pore cells.

GPC analysis (Table S3) confirmed that the pre‐curing step of the PDMS solution substantially increased the molecular weight, which in turn promoted the formation of a denser and more cohesive surface layer. This molecular weight enhancement is critical because higher‐Mw PDMS chains limit chain mobility, leading to tighter network packing and reduced free volume, thereby suppressing high molecular weight silicone oil uptake and enhancing selectivity. By contrast, direct dilution of Sylgard 184 without pre‐curing produced a much lower molecular weight and correspondingly looser PDMS surface, which resulted in a compromised barrier and markedly reduced separation performance. According to morphological analysis using SEM further supported these findings. Only the 10 wt% PDMS solution formed a well‐defined and uniform coating layer, while the 2 and 6 wt% solutions penetrated into the porous support without developing a dense selective layer (Figure S5). This indicates that both concentration and viscosity of the coating solution are pivotal to controlling surface coverage and preventing pore infiltration. In addition, optimization of the dipping time demonstrated that a 10 s exposure yielded the most favorable PDMS layer thickness, balancing transport resistance and selectivity, which directly translated into the highest separation factors in pure gas permeation tests (Figure S6). Under these optimized conditions, SEM revealed a uniform outer PDMS coating with a thickness of 3.35 µm (Figure 2), confirming that the dip‐coating process can reproducibly produce defect‐free selective layers suitable for sorp‐vection applications.

### Pure Gas Permeance and Separation Testing

The pure gas permeation tests, conducted with CO_2_ and N_2_, allowed determination of the selectivity for CO_2_/N_2_. Initial tests on fibers before PDMS coating showed CO_2_ permeance of about 1474.4 ± 1.8 GPU at 50 psia, with a selectivity of 1.2, suggesting that the gas transport in the uncoated fibers is mostly governed by Knudsen diffusion due to the relatively large pore size and lack of selective coating. While pure gas permeance and selectivity do not directly correlate with the actual sorp‐vection performance, they are useful for identifying defects in the fibers that could lead to leakage in the selective layer. After applying the PDMS layer, with varying ratios of base and curing agent, the dual‐layer separating fibers were tested to identify the most effective composition for sorp‐vection. The pure gas separation data shown in Table S4, indicated that all compositions achieved CO_2_/N_2_ selectivity close to that of pure PDMS dense flat films, typically around a factor of 11, with a CO_2_ permeance in the range of 300 GPU.^[^ ^41^ ^]^

### Sorp‐Vection Behavior in the Presence of CO_2_: Comparison with Either Pressure‐Driven Transport or N_2_ as Sorp‐Vection Agent

#### Separation Behavior Under D4 Rich Feed (85 wt% D4/Silicone Oil Mixture)

**Figure 3:**
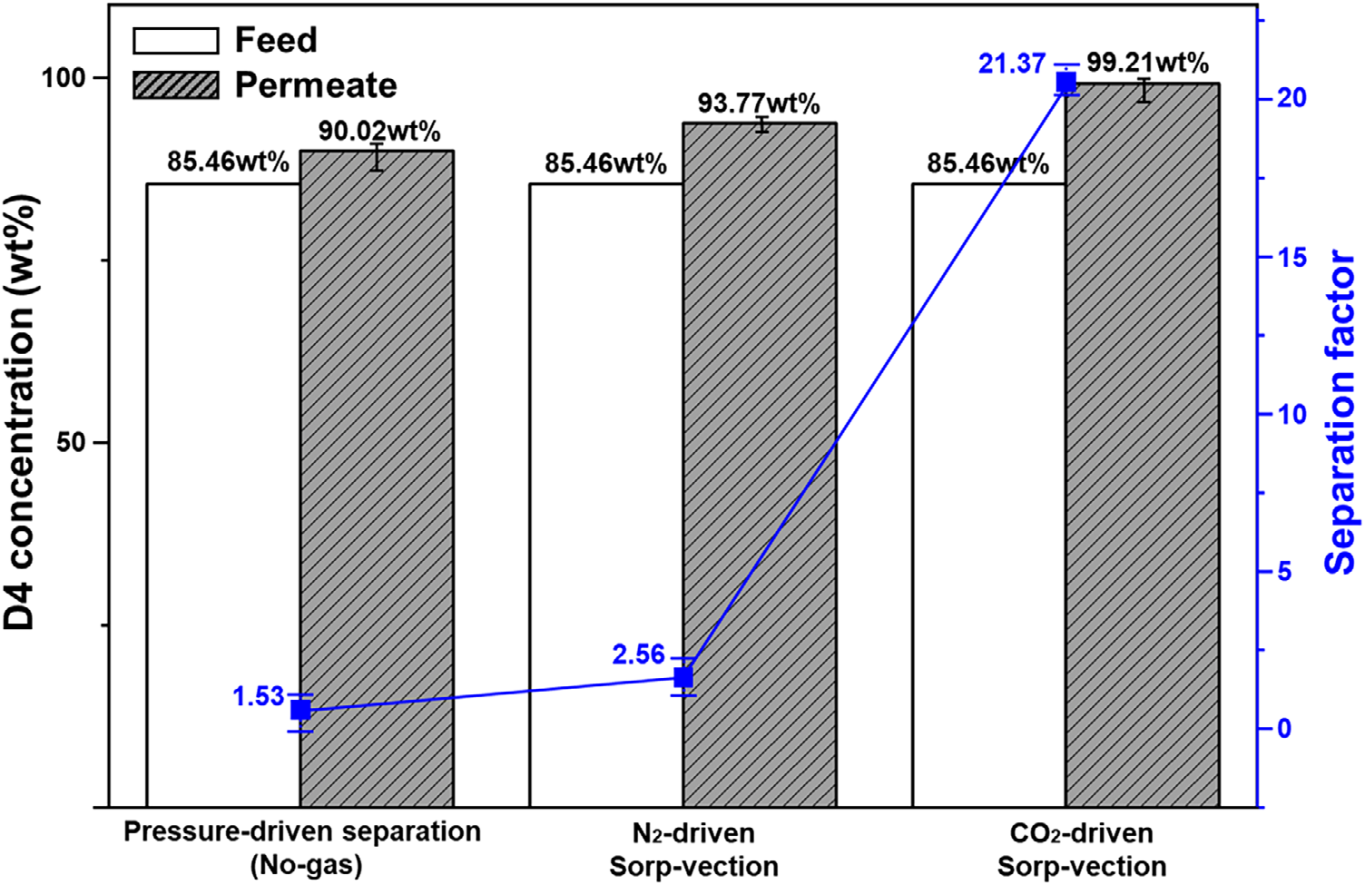
Comparison of separation performance: pressure‐driven reverse osmosis versus N_2_‐ and CO_2_‐driven sorp‐vection in D4 rich feed of 85wt% D4/silicone oil mixture. The measured fluxes were 0.11 ± 0.04 kg/(m^2^ hr) for pressure‐driven case, 0.12 ± 0.04 kg/(m^2^ hr) for N_2_‐driven sorp‐vection and 0.67 ± 0.13 kg/(m^2^ hr) for CO_2_‐driven sorp‐vection, respectively. Error bars represent standard deviations of three replicate experiments on the same module.

A comparative analysis between sorp‐vection and pressure‐driven separation (reverse osmosis) was conducted using different D4 ratio of D4/silicone oil mixture (85 and 30 wt%) to highlight the underlying transport mechanism features and demonstrate the advantages of sorp‐vection. All experiments were conducted at 500 psia, which was proven to be an attractive pressure of sorp‐vection as shown in Table S5 in Supporting information. Starting with 85% (rich) D4 silicone oil mixture, sorp‐vection separation was performed at 500 psia using CO_2_ as the driving gas. Also, pressure‐driven separation was tested under the same pressures using liquid‐only feed with no gas involvement. N_2_‐driven sorp‐vection testing was also conducted and under the same pressure of N_2_ gas. Figure 3 summarizes the selectivity results from three independent experiments conducted in triplicate with identical modules prepared under the same conditions, each exhibiting measurable permeation. This fact notwithstanding, the total flux for the “no gas” reverse osmosis case was very low and only approximately 0.11 ± 0.04 kg/(m^2^hr). In the pressure‐driven reverse osmosis (RO) experiment, the permeate concentration increased from 85.46wt% in the feed to 90.02 wt%, corresponding to a separation factor of 1.53. By contrast, sorp‐vection showed clear advantages. Although N_2_‐driven sorp‐vection yielded a modest separation factor of 2.56, CO_2_‐driven sorp‐vection achieved 21.37, which was approximately 14 times higher than conventional RO and over 8 times higher than the N_2_‐driven sorp‐vection case. The much higher permeability of CO_2_ versus N_2_ in the absence of either D4 or silicone oil was expected to give fluxes of both D4 and silicone oil for CO_2_ versus N_2_ sorp‐vection differing by a factor of 11, but with expected similar separation factors between the two compounds.^[^ ^42^ ^]^ N_2_‐driven sorp‐vection exhibits much lower performance because its permeability and sorption in PDMS are limited, resulting in low fluxes such that the term on the right‐hand side of Equation 8 becomes negligible. We speculate that this limitation also intensifies internal concentration polarization at the downstream of the PDMS selective layer, which reduces both the flux and the achievable separation factor compared to CO_2_.

During the sorp‐vection separation process, a decreasing CO_2_ flux was observed. The CO_2_ flux during the sorp‐vection process with 500 psia saturated 85% D4 rich feed, showed a decreased from 17.2 ± 3.8 kg CO_2_/(m^2^ hr) to a value of 5.7 ± 1.4 kg CO_2_/(m^2^ hr) within 20 min after starting the sorp‐vection experiment with 500 psia saturated 85% D4 in oil. So, it was not surprising to see a very low flux for N_2_ as well as for the RO case.

The D4 concentration in the permeate reached 99.21 wt% in CO_2_‐driven sorp‐vection experiment. In all cases, however, the effective fluxes were surprisingly low after allowing stabilization for up to 20 min. While this comparison highlights advantages of the sorp‐vection transport mechanism, particularly when CO_2_ is used as the driving gas it also reveals unanticipated problematic surprises and their causes—as well as paths to address these problems as discussed below.

#### Separation Behavior Under 30 wt% D4 Feed (30 wt% D4/Silicone Oil Mixture)

While 85 wt% D4 resulted in effective permeation across all three separation approaches: RO, N_2_‐driven, and CO_2_‐driven sorp‐vection, the advantages of sorp‐vection become significantly more obvious upon moving to 30 wt% feed D4 conditions. Conventional membrane separation methods exhibit critical limitations when processing higher solute concentration feed, and such limitations are evident even at the moderately diluted concentration of 30 wt% D4, which is not an extremely low concentration. This highlights the superior performance of sorp‐vection, even under conditions where traditional methods begin to falter.

Table 1 shows the results of RO and CO_2_‐driven sorp‐vection on the dilute feed of 30 wt% D4 in D4/silicone oil mixture at 500 psia. Each experiment was conducted in triplicate with identical modules prepared under the same conditions with the upstream pressure condition at 500 psia. The RO cannot effectively separate the D4 from the 30 wt% diluted feed of D4/silicone oil, since there was no practical permeate flow observed during the RO experiment at 500 psia feed for 14.7 psia permeate since the Δ*P* − Δπ = (Δ*P_eff_*)_*min*_. However, CO_2_‐driven sorp‐vection showed a practical permeate flow with a high separation factor that reaches about 13.63. The N_2_‐driven sorp‐vection also showed non‐measurable flux.

**Table 1:** Comparative analysis of RO, N_2_‐driven and CO_2_‐driven sorp‐vection separation in diluted D4 feed (30 wt% of D4 concentration in the D4/silicone oil mixture) at 500 psia.

Parameter	Pressure‐driven (RO)	N_2_‐driven sorp‐vection	CO_2_‐driven sorp‐vection
Operating Pressure	500 psia	500 psia	500 psia
Observed Flux	Not detected	Not detected	0.58 ± 0.22 kg m^−2^·hr
Separation factor	N/A	N/A	13.63 ± 0.13

**Figure 4:**
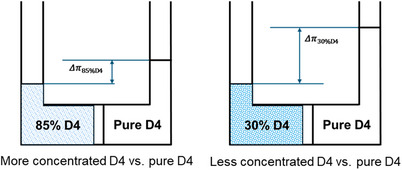
Comparison between Δπ of 85 and 30 wt% D4/silicone oil mixture. Δ*P* − Δπ = (Δ*P*_eff_)_min_, so, for Δ*P* = 500 psia,  (Δ*P*_eff_)_85%*D*4_ >  (Δ*P*_eff_)_30%*D*4_ and if Δ*P* − Δπ = (Δ*P*_eff_)_min_ = 0, no flux occurs for RO case for 30% D4 case. Note that the pure D4 would be in the liquid state in both of cases at the permeation side. (Calculated using Van't Hoff equation).

The failure of pressure‐driven RO separation in diluted D4 feed (30wt% D4 feed) at 500 psia can be attributed to an insufficient net effective driving force to overcome the chemical potential in the permeating species (D4) differences between the feed and permeate that impose excessive requirements on applied feed pressure in the RO modality. As shown in Figure 4, while the permeate side is ambient air at the start of the experiment, once sorp‐vection occurs, the permeate stream becomes highly enriched in D4 (measured at ∼95 wt% in our experiments). To provide a tractable estimate of the osmotic driving force, we approximated the permeate side as pure D4 when calculating Δπ. This approximation slightly overestimates the practical pressure difference, but it captures the key trend. With this framework, the situation differs from a simple concentration‐gradient picture. If the permeate is assumed to be D4‐rich, the effective Δπ is larger for the 30 wt% feed than for the 85 wt% feed, which explains why separation is more difficult at lower concentrations.

In contrast, the CO_2_‐driven sorp‐vection separation demonstrated successful performance under downstream pressure conditions, achieving a permeate flux of 0.58 kg m^−^^2^·h and a separation factor of 13.63 for the same 30% D4 feed solution, well below the required minimum effective reverse osmosis pressure needed for 30% D4 feed. This shows that the advantages of sorp‐vection mechanism relies on the preferential partitioning of D4 into the membrane phase and is enabled by a CO_2_ convective flow to carry the D4 across the membrane. This convective assistance enables separation under moderate pressures (e.g., 500 psia), where traditional pressure‐driven methods such as reverse osmosis typically fail due to an insufficient net chemical potential driving force. The distinction in effective pressure requirements between RO and sorp‐vection becomes even more critical in highly dilute D4 feeds, such as 1–10 wt% D4/silicone oil mixtures. Rather than solely overcoming the chemical potential barriers through transmembrane pressure, sorp‐vection benefits from the appealing convective CO_2_ flow, which enables sustained flux and high separation performance as compared to other gases in Figure S8. Fluxes would be unattractive, since small but non‐zero quantities of oil permeate across the selective layer and clog the Torlon support. Fortunately, besides convectively motivating D4 through the selective layer, by operating with a vacuum on the fiber bore the permeated CO_2_ volume leaving the downstream face of the *selective layer* expands greatly to physically flush both D4 and oil into the bore.

### Sorp‐Vection Testing Predictions Based on Sorption

The sorption behavior of the cyclic siloxane molecule D4 and silicone oil purchased from Sigma Aldrich was examined through liquid swelling experiments on dense PDMS film conducted at room temperature (25 °C). The liquid swelling experiments were repeated in triplicate on three separate films. Sorption results, expressed as the uptake of solvent per unit mass of dry polymer film, were used to theoretically estimate the sorp‐vection selectivity results.

**Figure 5:**
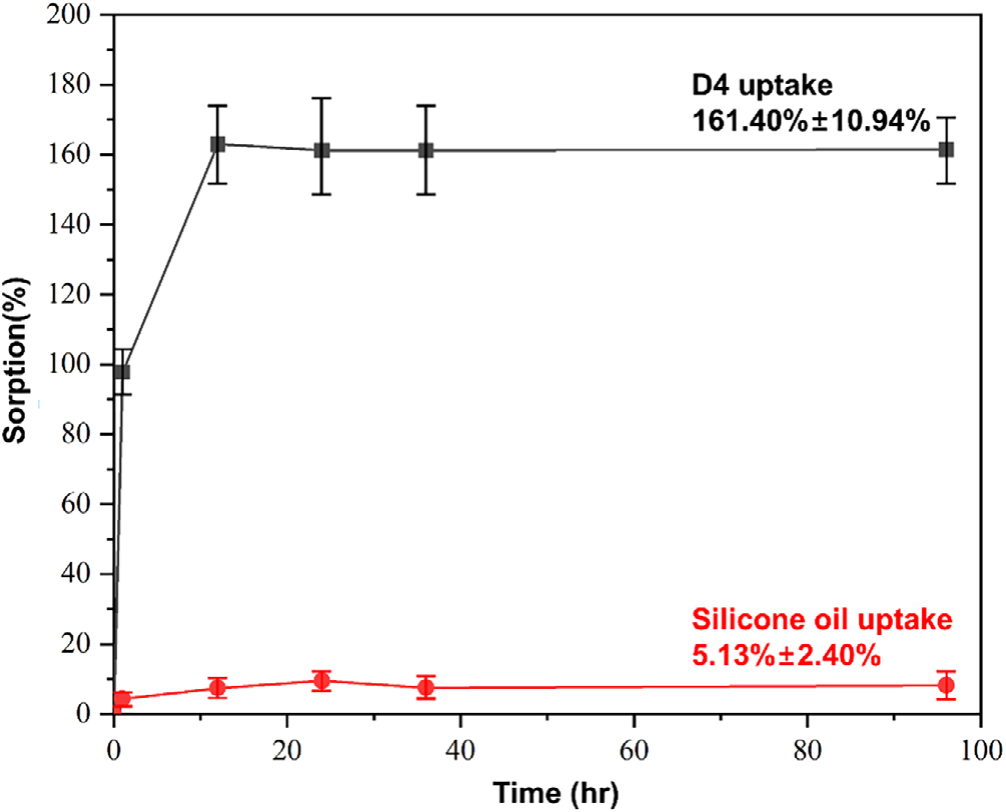
Liquid sorption testing on PDMS film at 25 °C.

As illustrated in the liquid sorption testing results (Figure 5), the sorption dynamics of the PDMS film were tested. The results show that pure D4 uptake is about 161.40% ± 10.94%, but for pure silicone oil, it is just about 5.13% ± 2.40%.

Based on these observations, the experimental sorp‐vection data can be understood using the simple theoretical model previously discussed in the Theory section, and these predictions will be further elaborated upon in the sorp‐vection results.

With the equation for sorp‐vection separation factor suggested in the Theory section, the separation factor is derived from the calculation of flux of Oil, D4, and CO_2_. Using Equation (7), and the sorption data of CO_2_,^[^ ^12^ ^]^ and the measured P/l = 300 GPU (described in Pure gas separation test section, Supporting Information), we can estimate the flux of the CO_2_ as:

(10) $$\begin{array}{cc} n_{CO2}=\left(\frac{P}{l}\right)\;\left[\Delta p_{CO2}\right] & =300\times 10^{-6}\frac{486}{14.7}\left[76\right]\;\left[\frac{44}{22400}\right]\\ & \;=0.00148\frac{g}{cm^{2}s} \end{array}$$

As A stands for D4, and *C* for silicone oil, the conventional diffusion flux can be neglected but bulk flow contribution should be included as Equations (3)–(5). For these components, the equation for separation factors can be simplified using the following expressions for oil and D4 flux:

(11) $$n_{Oil}=\omega_{Oil,\;m}\;\left(n_{Oil}+n_{D4}+0.00148g/cm^{2}s\right)$$

(12) $$n_{D4}=\omega_{D4,m}\;\left(n_{Oil}+n_{D4}+0.00148g/cm^{2}s\right)$$

Using the sorption results from PDMS films in Figure 5, we can estimate the weight fraction of the oil and D4. Next, the flux of oil and D4 can be estimated by using Equations (11) and (12). Using the sorption data presented in Figure 5, the theoretically estimated separation factor is calculated to be 12.96 based on Equations (11) and (12), together with the separation factor definition in Equation (9) as described in Section S1. According to these equations, the separation factor is primarily governed by the sorption isotherms of D4 and silicone oil and should be mostly independent of the feed concentration. These equations demonstrate that the separation factor is strongly influenced by the sorption behavior of silicone oil in the membrane. Specifically, a higher cross‐linking density in the PDMS structure leads to greater exclusion of silicone oil, thereby resulting in a higher separation factor. The results indicate that oil exclusion plays a critical role in enhancing separation performance. So, increasing the cross‐linking density of the PDMS selective layer is essential for minimizing silicone oil uptake and achieving high separation factors.

Besides the obvious fundamental factors, additional factors such as viscosity and concentration polarization may still influence the observed separation factor in practice. In fact, the issue related to the viscosity of the oil and D4 in the pores of the Torlon supporting the PDMS selective layer is crucial. Indeed, if not handled properly, the “selective layer” will grow as the high viscosity silicone oil (with properties similar to PDMS) will cause an effective thickening of the “selective layer” and reduce CO_2_ permeance and retard the passage of the permeate D4 and oil into the fiber bore for removal.

### Sorp‐Vection System Performance Analysis on Sigma Aldrich Silicone Oil Based Feed

**Figure 6:**
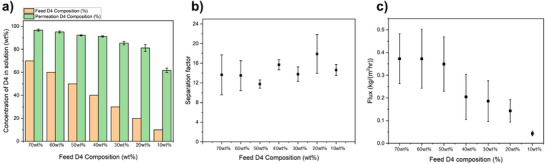
Sorp‐vection testing results at 500 psia CO_2_ for different concentration feed based on Sigma‐Aldrich oil. a) Feed D4 composition and D4 composition in the permeation, b) Separation factor for sorp‐vection testing on feed with different D4 composition from 70 to 10 wt%, c) Permeation flux observation on sorp‐vection testing tested with different D4 composition feed. Error bars represent standard deviations of three replicate experiments on the same module.

After validating the pure gas separation performance, to ensure that the PDMS layer was uniformly dip‐coated on the hollow fiber surface without defects, the dual‐layer hollow fiber modules were used in the sorp‐vection system for separating D4 from the silicone oil mixture. Prior to testing with industrial‐grade silicone oil, we first used Sigma Aldrich silicone oil feed, which contains no additional contaminants and has a similar viscosity and molecular weight to our target silicone oil from Dow Chemical to establish baseline data for a theoretical sorp‐vection case study. We initiated testing with a D4‐rich binary composition of 70 wt%/30 wt% D4/Silicone oil and used different concentrations of D4 composition from 70 to 10 wt% in the mixture to observe the effect and efficiency of sorp‐vection separation in the silicone oil mixture from high to low concentration contaminant in the mixture. During the sorp‐vection experiments, a minimum CO_2_ pressure of 500 psia was identified as optimal, providing the highest separation factor along with sufficient permeate flux as shown in Table S5. Prior to testing, all silicone oil mixtures with varying D4 compositions were fully saturated with CO_2_ at 500 psia to ensure adequate driving force for sorp‐vection separation. Each experiment was conducted in triplicate with identical modules prepared under the same conditions, with the upstream pressure condition at 500 psia. As shown in Figure 6, the separation factors remained consistently across a wide range of feed concentrations, from 70 wt% (rich feed) to 10wt% (dilute feed). In Figure 6a, the D4 composition in the feed is compared with that in the permeate. The D4 concentration in the permeate was consistently higher than in the corresponding feed, exceeding 90 wt% for all feed mixtures containing more than 40 wt% D4. This result underscores the high efficiency of the sorp‐vection technique in selectively permeating a diluted compound. Figure 6b presents the separation factors for each tested feed with varying D4 compositions. Within experimental uncertainty, the stable separation factor is observed as the feed D4 concentration decreased from 70 to 10 wt%, remaining within the range of 13–17. This is similar to the estimated separation factor calculated in Section “Sorp‐Vection Testing Predictions Based on Sorption”. In contrast, the measured permeation flux showed a monotonic decreasing trend, with the highest flux observed at 70 wt% D4 (approximately 0.37 kg m^−^^2^·hr), dropping to 0.04 kg m^−^^2^·hr at 10 wt% D4.

The observed decrease in flux is likely attributed to the growing impact of concentration polarization and accumulation of silicone oil with elevated viscosity at the downstream face of the PDMS selective layer. Both phenomena hinder the permeation across the PDMS selective layer in the sorp‐vection process. As shown in Figure S9, the viscosity of D4 and silicone oil mixture exhibits a nonlinear increase as the D4 concentration decreases, with significant changes occurring around 20 and 50 wt%. These may correspond to the oil chain entanglement changes within the mixture. This viscosity trend aligns well with the decreasing flux trend, which shows a marked decline near D4 concentration of 50 and 10 wt%. For the sorp‐vection case, a buildup of silicone oil also can occur at the downstream face of the *selective layer*. We speculate that this is the main reason for the decreasing flux with the stable separation factor, as separation factor is only related to the weight fraction of the D4 according to the Equation (9). On the other hand, the flux is influenced by the buildup of silicone oil at the PDMS downstream face, which also hinders the CO_2_ movement across the membrane. This factor can be investigated but is not the main focus of this proof‐of‐concept study. In any case, a more specialized form of “internal concentration polarization” is active and is indirectly overcome by the CO_2_ that exits at the downstream face of the selective layer. This CO_2_ flushes all permeate into the bore.

The stability of the separation factor across different D4 feed ratios is attributed to the consistent low sorption rate of silicone oil within the membrane layer. According to Equations (6)–(8), the main driving force for sorp‐vection process is the flux of CO_2_, and the flux of each component (*n*_A_ and *n*_C_) can be described as the product of its weight fraction and the CO_2_ flux, which acts as the sorp‐vection driving force. This transport mechanism explains why the separation factor remains stable even when the D4 concentration in the feed varies. Since the sorption of silicone oil does not change significantly, only the weight fraction of D4 varies, which caused an overall decrease in permeation flux. However, according to Equation (9), the separation factor depends solely on the relative weight fractions of components A and C in the membrane divided by the concentration of the feed, which accounts for the observed stability. This consistent separation factor across varying feed compositions further supports the theoretical foundation of the sorp‐vection mechanism.

### Sorp‐Vection System Performance Analysis on Industrial Grade Silicone Oil

Building upon the baseline experiments conducted with high‐purity silicone oil from Sigma‐Aldrich, which is free of any contaminant, we advanced our investigation to more complex conditions using an industrial‐grade silicone oil known as DOW‐SFD (SFD). This transition marks a critical step in evaluating the practical applicability of the sorp‐vection process for real‐world silicone oil purification, where feedstocks often contain impurities and process byproducts.

As indicated in Table 2, sorp‐vection testing was carried out using industrial‐grade silicone oil. For the 8.5 wt% D4/SFD feed, the separation factor reached 17.46 under standard conditions, demonstrating the technique's effectiveness even in more challenging feed compositions.

**Table 2:** CO_2_‐driven Sorp‐vection separation in diluted D4 feed with industrial grade silicone oil (8.5 wt% of D4 concentration in the D4/DOW‐SFD) at 500 psia. (Error bars represent testing of different sorp‐vection tests (*N* = 3).)

Pressure	Feed (D4 wt%)	Permeate (D4 wt%)	Separation factor	Flux
500 psia	8.50%	63.53% ± 0.02%	19.05 ± 2.29	0.05 ± 0.01 kg/m^−2^ hr

Theoretical predictions based on Equations (11) and (12) in Section “Sorp‐Vection Testing Predictions Based on Sorption” estimated a separation factor of 17.58 for an 8.5 wt% D4 feed, closely matching the experimental value of 19.05 for the 500 psia case. This agreement validates the simple and useful predictive strength of the proposed sorp‐vection framework.

Furthermore, the consistent performance of sorp‐vection with industrial‐grade silicone oil compared to the results from pure silicone oil confirms the relevance and robustness of the system. Clearly, the second stage sorp‐vection module can raise the final product D4 to >95 wt%. Overall, these findings reinforce the potential of sorp‐vection as a next‐generation purification platform capable of meeting industrial demands under realistic and challenging conditions.

### Overcoming “Internal Polarization” by Modifying the Sorp‐Vection Platform

**Figure 7:**
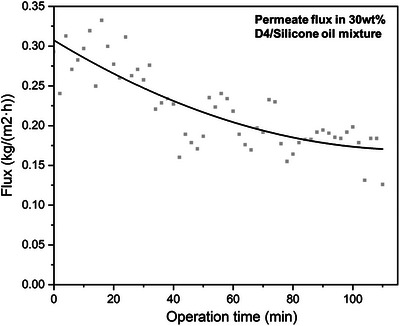
Permeate flux during CO_2_‐driven sorp‐vection process (tested with 30 wt% D4 in silicone oil mixture).

A major set of insights arose from the above proof‐of‐concept work, which clearly shows a simple modification that should provide high flux and stable operation. Specifically, Figure 7 shows a time record of the flux determined for the 30% D4/70% silicone oil feed that resulted in the reported value for this feed in Figure 6. The surprising precipitous drop to the recorded value reported in Figure 7 was eye‐opening to us, and we hypothesized that the drop in the flux was due to the phenomenon mentioned above that we can refer to as “internal concentration polarization”.^[^ ^43^, ^44^, ^45^ ^]^ Specifically, the fine‐pored support in Figure 2c could steadily fill up with the small but non‐zero high viscosity oil and progressively create a secondary non‐crosslinked PDMS selective layer. Although the CO_2_ permeability of such a layer will not be identical to that of the actual 3.35 µm crosslinked selective layer, it will be similar and eventually add another 150 µm resistance. This means that “actual” membrane resistance is 50 times thicker than the one we envisioned being in place. We also realized that if we pulled a vacuum on the fiber bore, when CO_2_ emerges from the membrane downstream, it will undergo *a flash expansion* and displace the D4 and especially the oil filling the pores next to the bottom of the PDMS membrane selective layer. This desirable outcome will provide an in‐situ sweep to keep the support pores open. We are in the process of systematically testing this concept, and it will be the topic of a subsequent paper.

## Conclusion

By introducing sorp‐vection as a new framework for membrane separations with coupled sorption and gas permeation, this study demonstrates a practical realization of an unconventional transport mechanism in which high gas fluxes directly couple with sorbed solutes within a dense polymer layer. This approach enables highly selective and energy‐efficient method for separating low molecular weight cyclic siloxanes from silicone oil. A dual‐layer with a PDMS coating porous hollow fiber membrane was developed and characterized, representing a significant advancement in membrane technology. The PDMS coating enabled selective absorption of low molecular weight siloxanes while effectively rejecting higher molecular weight silicone oil components, thereby ensuring product purity.

Consistent separation performance was achieved even at high concentrations of D4, with separation factors ranging from 12.5 to 17.5 for feeds containing 10%–70% cyclic content. Notably, a separation factor of 17.46 was attained with industrial‐grade silicone oil containing only 8.5% D4, further validating the robustness of the process. This result is particularly significant considering the main industrial demand lies in purifying silicone oils with low D4 concentrations, often down to 1–2 wt%, which poses considerable separation challenges. Achieving strong separation performance at the 10 wt% feed level, therefore, represents a meaningful outcome of this study.

The close correlation between theoretical predictions and experimental outcomes not only confirms the effectiveness of sorp‐vection but also reinforces its potential as a transformative separation strategy. These results suggest that sorp‐vection could be extended beyond silicone oil purification, offering a broadly applicable, non‐thermal solution to separation challenges across various industrial sectors. We also have a proposed mode of operation for sorp‐vection processes with a vacuum on the fiber bore that we are hopeful will result in even higher permeance of modules, particularly at low D4 concentrations. Strategies such as increasing CO_2_ pressure and employing multi‐fiber configurations are expected to enhance performance further, and these will be reported after careful testing.

## Conflict of Interests

The authors declare no conflict of interest.

## Supporting information



Supporting Information

## Data Availability

The data that support the findings of this study are available in the Supporting Information of this article.
