# Supplementary material for: Sorp‐Vection‐Based Membrane Silicone Oil Purification
Source: Angew Chem Int Ed Engl. 2025 Nov 12;65(2):e202516848. doi: 10.1002/anie.202516848 (PMC12790354; doi:10.1002/anie.202516848)
Supplement: Supplementary file 1 — Supporting Information [file ANIE-65-e202516848-s001.docx]

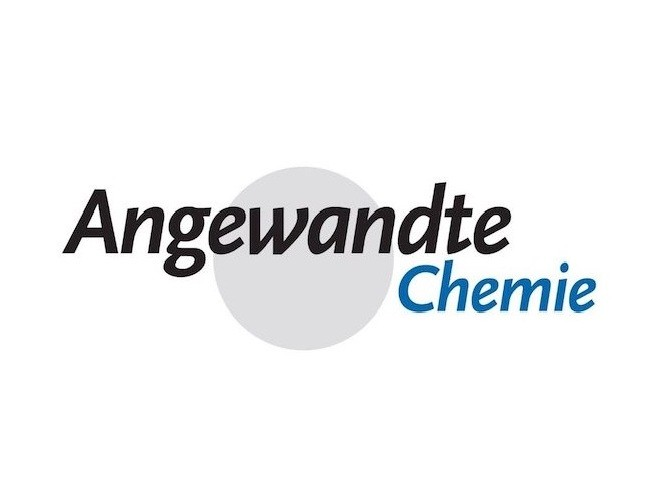


Sorp-vection-Based Membrane Silicone Oil Purification

Jinyoung Kim^1^, Yuhe Cao^1^, Wulin Qiu^1^, Zhongyun Liu^1^, Steven Schlosser^1^, Reza Haghpanah^2^, Dimitris Katsoulis^2^, Jay Rose^2^, Seo-Yul Kim^1^, Hammed A. Balogun^1^, Ryan Lively^1^ and William J. Koros^1*^

**Table of Contents**

[**Materials** 3](#_Toc204218040)

[***Figure S1.*** Sorp-vection system demonstration. (a) Schematic of the dual-layer hollow fiber module. (b) Schematic of the sorp-vection system 5](#_Toc204218040)

[***Scheme S1.*** Chemical structure of Octamethylcyclotetrasiloxane(D4), Decamethylcyclopentasiloxane (D5), Dodecamethylcyclohexasiloxane (D6) 7](#_Toc204218040)

[***Figure S2.*** Fiber spinning system and procedures 7](#_Toc204218042)

[***Figure S3.*** CO2 saturation process before sorp-vection process. 8](#_Toc204218043)

[***Figure S4.*** Torlon hollow fiber SEM image, A) State #4 fibers with an outer diameter of approximately 615 μm with a wall thickness of ~120 μm, B) State #5 fibers with outer diameter of ~500 μm and a thicker wall of ~150 μm. 8](#_Toc204218044)

[***Figure*** ***S5.*** PDMS layer on the Torlon hollow fiber dip-coated with different concentration of PDMS in Heptane coated with solution of (A) 2wt% PDMS in Heptane, (B) 6wt% PDMS in Heptane, (C)10wt% PDMS in Heptane. 9](#_Toc204218046)

[***Figure S6.*** Thickness of PDMS layer on the Torlon hollow fiber with different dip-coating time in PDMS solution (A) 2 seconds dip-coated layer with thickness of 863nm, (B) 6 seconds dip-coated layer with thickness of 2.81μm, and (C) 10 seconds dip-coated layer with thickness of 4.15μm. 10](#_Toc204218047)

[***Figure S7.*** Minimum effective pressure required for separation, estimated from chemical potential differences based on D4 activity gradients across the membrane. 11](#_Toc204218048)

[***Figure S8.*** Solubility of different gases in PDMS and silicone oil ^[1,2]^ 12](#_Toc204218049)

[Calculation of separation factor based on Sorp-vection theory. 13](#_Toc204218050)

[***Table S1.*** Fiber spinning conditions for state number #1 to #9. 15](#_Toc204218051)

[***Table S2.*** Pure gas permeation test on fiber state number #1 to #9. 16](#_Toc204218052)

[***Table*** ***S3.*** Comparison of PDMS Molecular Weight: Pre-Cured vs. Non-Cured. 17](#_Toc204218053)

[***Table S4.*** Pure gas permeation test on PDMS coated fiber on fiber state #4-5. 17](#_Toc204218054)

[***Table S5.*** CO_2_-driven sorp-vection performance at different feed pressures 18](#_Toc204218054)

[***Reference*** : 18](#_Toc204218055)

Materials

For preliminary sorp-vection testing, octamethylcyclotetrasiloxane (D4) and silicone oil purchased from Sigma Aldrich were used. The polydimethylsiloxane (PDMS) base polymer and curing agent, part of the Sylgard 184 silicone elastomer kit, were procured from Dow Corning.

Porous hollow fiber were fabricated to serve as the support structures for the PDMS membranes. Torlon was chosen as the material for the porous supports due to its chemical resistance and low levels of dilation in the presence of D4 and the silicone oil. In the fabrication of hollow Torlon fibers, polyamide-imide (PAI) (Torlon®, Solvay Advanced Polymers®) and poly(vinylpyrrolidone) (PVP) (MW 55,000, Sigma-Aldrich) were to form the hollow fibers. Prior to use, all polymers were vacuum dried at 110°C for one day to eliminate moisture. n-Methyl-2-pyrrolidone (NMP) (Reagent Plus 99%, Sigma-Aldrich) served as the solvent in the polymer-spinning dope. Methanol (MeOH) (99.8%, ACS Reagent, Sigma-Aldrich), n-heptane (anhydrous, 99%, ACS Reagent, Sigma-Aldrich), and hexanes (ACS Reagent, >98.5%, Baker) were employed in the solvent exchange stage of the fiber formation process, after spinning. All solvents and non-solvents were used as received, without further purification or modification. All fittings used for module making were purchased from Swagelok® Georgia.

Fabrication of Hollow Torlon Fiber Substrate

Asymmetric hollow fiber membranes were produced using a dry-jet/wet-quench spinning process. In this method, a polymer solution (dope) and a bore fluid were coextruded through a spinneret. Initially, the fibers entered an air gap (dry-jet) and subsequently were immersed into an aqueous quench bath (wet-quench). During this transition, water (non-solvent) diffused into the nascent fibers, inducing phase separation in the underlying substrate, and leading to the formation of a porous substructure while preventing excessive porosity in the skin. The bore of the hollow fiber was formed by simultaneous extrusion of the bore fluid with the dope. This fabrication process has been described in detail in our previous study.^[36]^ The dry-jet/wet-quench process for producing precursor hollow fiber membranes and the optimized composition of the dope is described in detail in Figure. S2.

The spinning dopes were prepared in sealed glass bottles and dissolved on a roller under a heat lamp (50°C) until homogeneous. After cooling to room temperature, the dope was transferred to a 500ml syringe pump to degas for 24 hours at 50°C. Post-spinning, the fibers were immersed in water for several days to eliminate residual solvent traces, followed by sequential one-hour solvent exchange baths of methanol and hexane. Finally, the fibers were air-dried for one hour at room temperature and subsequently dried under vacuum at 75–85°C for three hours.

As noted in the manuscript, Torlon hollow fibers comprise the inner supporting structure of the separation fibers dip-coated with the PDMS layer on the surface. The Torlon hollow fibers were fabricated with a uniform cross-sectional porous structure to support the selective PDMS. This feature will be discussed later, since we discovered that this positive feature can also result in a subtle secondary resistance build up in the tight pore structure of the membrane if it is not managed properly. The Torlon hollow fiber is fabricated using the “dry-jet”/ “wet-quench” method in the spinning system (Figure S2), using a dope solution and a bore fluid to form a core layer and bore-side of the fibers, respectively. Nine different states of fiber were fabricated and tested with pure gas permeance tests (Table S1-2) and the most uniform and stable structure fiber state (State #4 and #5) was selected for this study. Visual uniformity of fibers was also observed through Scanning Electron Microscopy (SEM) analysis, which revealed well-defined circular bores and porous support comprising the support wall that was roughly 150 μm in thickness (Figure S4). The outer diameter (OD) and wall thickness were consistent in both fiber states. As noted earlier, our subsequent analysis suggests the uniformly distributed, relatively tight porous support structure for the dense PDMS selective layer was not optimum. Unlike the case for a simple sorption-diffusion gas separation asymmetric membrane, the porous support needs a more advanced approach to be optimum for sorp-vection, especially for a feed comprised of a viscous oil like that studied here. In any case, independent of the porous support, formation of a high integrity PDMS selectivity is crucial and is discussed next.

PDMS Dip-Coating Process

Following fiber preparation, a key step involves the application of a PDMS layer to form a separating layer. The PDMS layer acts as the selective layer, making the preparation of the PDMS dipping solution and the dipping process important for optimal performance. PDMS solutions were formulated using the base polymer and curing agent from the Sylgard 184 kit, with the ratio of base to curing agent meticulously controlled to optimize the properties of the dual-layer fibers. Here, we use the optimized base to curing agent ratio of 5:1 in the reaction.

The mixing process began by blending the base and curing agent at a predefined ratio, followed by vortex mixing at 3000 rpm for 1 minute. This mixture was then heated to 90°C for 7 minutes to facilitate proper polymerization. Subsequently, the solution was diluted with heptane to create a coating solution, optimized for dip-coating, with a concentration of 2–10 wt% polymer. After resting at room temperature for 24 hours, viscosity and polymerization checks were conducted to ensure the solution was suitable for coating the fibers. A bubble-free, homogeneous coating solution was achieved after 24 hours. The dip-coating method was then employed to apply this solution onto the outer surface of the hollow fibers, with precise timing described in section 4.2 in the manuscript to ensure defect-free condition of the PDMS layer in this proof-of-concept study.

Torlon (polyamide-imide (PAI) hollow fibers serve as the supporting substrate for the dual-layer separating fiber in the sorp-vection process. The outer PDMS layer on the hollow fiber membranes acts as the selective entity that enables preferential permeation of cyclic siloxanes, component A, relative to the high molecular weight oil, component C. The separation performance of the PDMS layer is critically dependent on multiple factors including the layer's uniformity, thickness, crosslinking density, and precursor molecular weight. Among these factors, the two most influential factors are the thickness and crosslinking density of the PDMS layer. The thickness of the PDMS layer plays a key role in determining the permeation rate. A thicker layer increases the diffusion path length, which reduces the flux, while a thinner layer facilitates higher permeation rates due to shorter transport distances and lower resistance, but defect free properties are also key. The PDMS layer was coated on the fibers through a dipping process of the fibers into the diluted solution of PDMS solution composed of base solution and curing agent of Sylgard® 184, and the reaction between base solution and curing agent is shown in Scheme. S1. Since the viscosity of mixed solutions of base and curing agents is too high to achieve proper thickness of PDMS layer on the fiber, diluted PDMS is needed for the dipping process. Various solvents were tested to assess the solubility and affinity to both fibers and the PDMS solution. Among the solvents tested, the most appropriate solvent was found to be n-heptane for uniformly dip-coating on the fiber, and a proper swelling ratio of the solvent without affecting the supporting substrate of Torlon fibers. Prior to dilution of PDMS solution, a seven-minute curing step was introduced to pre-crosslink the PDMS in the dip-coating process. This step resulted in an increased molecular weight as confirmed by GPC analysis (Table S3) and to achieve an efficient separating layer, several factors for PDMS layer fabrication were varied. Molecular weight and crosslinking density of the PDMS, which determines the tightness of the PDMS surface layer, also serves as a critical factor for selectivity by suppressing silicone oil uptake. Direct dilution in n-heptane of Sylgard 184 without post-curing was also tested to assess the effect of molecular weight. Post-curing of the PDMS solution has proven to form a much higher molecular weight PDMS layer with tighter surface, and in turn a dramatic increase in both pure gas and sorp-vection separation factors. The concentration of PDMS diluted in n-heptane was preferred based on testing 2wt%, 6wt% and 10wt% PDMS solutions. After dip-coating, the 10 wt% PDMS solution formed a well-defined layer, as observed by SEM, whereas the 2 wt% and 6 wt% PDMS solutions penetrated into the porous surface without forming a dense PDMS layer (Figure S5). The dipping time of the hollow fiber into the PDMS dipping solution also influences the thickness of the PDMS layer on the surface. To optimize this factor, various dipping times (2, 6 and 10 seconds) were also investigated to determine the best dipping time for the highest separation factors in pure gas permeation tests. We found that the 10s dip-coating process gave the best thickness for sorp-vection layer (Figure S6). These studies allowed us to identify optimized conditions to achieve the preferred viscosity of the PDMS diluted solution and the required exposure time to achieve a layer that is neither too thick to impede the feed flow nor too thin to compromise selectivity. The optimal coating conditions were determined through systematic experiments, resulting in the use of a 10% concentration of PDMS solution and a dipping time of 10 seconds.

Morphological Characterization

Morphological characterization of the monolith hollow fibers was conducted using a Scanning Electron Microscope (SEM) (Hitachi SU8230, Japan) to analyze the cross-sectional architecture and overall fiber morphology. To prepare the samples, the fibers were sectioned using a cryo-microtome under liquid nitrogen to preserve the integrity of the PDMS layer and the underlying hollow fiber structure. The sections were then mounted on carbon tape and affixed to SEM stubs suitable for examination. Prior to imaging, the samples were sputter-coated with a thin layer of gold to prevent charging under the electron beam, ensuring high-resolution imaging. This process verified the uniformity and completeness of the PDMS layer formation on the hollow fibers, confirming their suitability for intended separation applications.

Pure Gas Separation Testing

PDMS-dip-coated hollow fibers were then made into sorp-vection modules, and all modules were initially assessed using pure component flux tests for N_2_ and CO_2_ at 35 °C using a constant-pressure system like that reported earlier to confirm the integrity of the PDMS layer on the hollow fiber substrate. This evaluation both supplements the morphological examination and addresses potential subtle defects that could affect performance. The PDMS dipping process not only adds a selective layer but also acts as a seal for pinhole defects in the fiber skin, effectively preventing minor surface flaws and minimizing Knudsen diffusion through these defects.^[2,39]^ Carbon dioxide (CO_2_) and nitrogen (N_2_) permeances were measured under a constant feed pressure of 50 psi, introduced to the shell-side of the hollow fiber membranes, while maintaining a bore-side pressure of 1 atm. The permeate flow was measured using a bubble flow meter once a steady state was achieved, facilitating the calculation of selectivity. Multiple replicates were conducted under all specified conditions to ensure the reproducibility of data and the permeance ($\mathbb{P}\mathcal{/l}$) was calculated using the following Equation (S1):

$$\begin{aligned} \frac{\mathbb{P}}{\mathcal{l}}={10}^{-6}\cdot\frac{Q_{p}\cdot52.83}{A\cdot T\cdot\Delta p}\#\left( S1 \right) \end{aligned}$$

where $\mathbb{P}$ is the gas permeability through the membrane. $\mathbb{P}\mathcal{/l}$ has units of “GPU”, or gas permeation units, which corresponds to 10^-6^ cm^3^(STP) cm^-2^ s^-1^ cmHg^-1^. In Equation (S1), $Q_{p}$ is permeate flow rate, given in milliliters per second (mL/sec); A is the active membrane area, specified in square centimeters (cm²); T represents the room temperature in Kelvin; and Δp is the pure gas transmembrane pressure difference, expressed in cm Hg.

The selectivity, denoted as $\alpha_{ij}$, quantifies the ideal efficiency of membrane separation for a pair of gases, defined by the ratio of the permeance of the faster permeating gas (𝑖) to that of the slower permeating gas (𝑗), as shown in Equation (S2):

$$\begin{aligned} \alpha_{ij}=\frac{\mathbb{P}_{i}\mathcal{/l}}{\mathbb{P}_{j}\mathcal{/l}}\#\left( S2 \right) \end{aligned}$$

Liquid Sorption Measurements

Sorption kinetics at unit activity were studied using dense PDMS films approximately 1mm thick. These films were submerged in pure liquid D4, pure silicone oil (Sigma-Aldrich) and industrial grade silicone oil (DOW-SFD), each contained within air-tight vials. The mass change between the swollen and dry films was monitored at predefined intervals until equilibrium was reached. At each designated weight recording point, the sample was removed from the vial and quickly blotted to remove any residual liquid on the external surface before the weight was measured. Each weighing was conducted in triplicate for each film to ensure accuracy.

Viscosity analysis on D4/silicone oil mixture with different D4 concentration

To investigate the viscosity behavior of D4/silicone oil mixtures across different compositions, a Brookfield rotational viscometer (Model DV-II+ Pro, Brookfield Engineering Laboratories, USA) was employed. Viscosity measurements were carried out at ambient temperature using the RV-5 spindle operated at a constant rotational speed of 50 rpm. Prior to measurement, samples were prepared by thorough mixing to ensure homogeneity and were then loaded into the standard Brookfield cylindrical sample chamber. Each measurement was recorded after the system reached steady-state rotation to minimize transient effects. The viscosity values were monitored as a function of D4 content ranging from 10 to 90 wt%, and the resulting data were used to assess the influence of composition on the rheological characteristics of the mixtures.

Sorp-vection System Setup and Analysis

As shown in Figure S1(a), the PDMS-coated hollow fiber was used to make membrane modules for the sorp-vection separation tests. As illustrated in Figure S1(b), the sorp-vection system incorporates two syringe pumps: one designated as the feed pump and the other as the collecting pump. The feed pump is responsible for delivering the CO_2_-saturated feed solution to the separating module. The retentate stream exiting the module is then directed to the collecting pump, which serves to collect the retentate after passage through the module. As noted above, CO_2_ plays the crucial role in achieving a desirable steady state permeation within the system and is the primary driving force for the sorp-vection separation process. This gas aids in the transport of cyclic siloxanes across the PDMS layer due to its permeability and solubility characteristics.


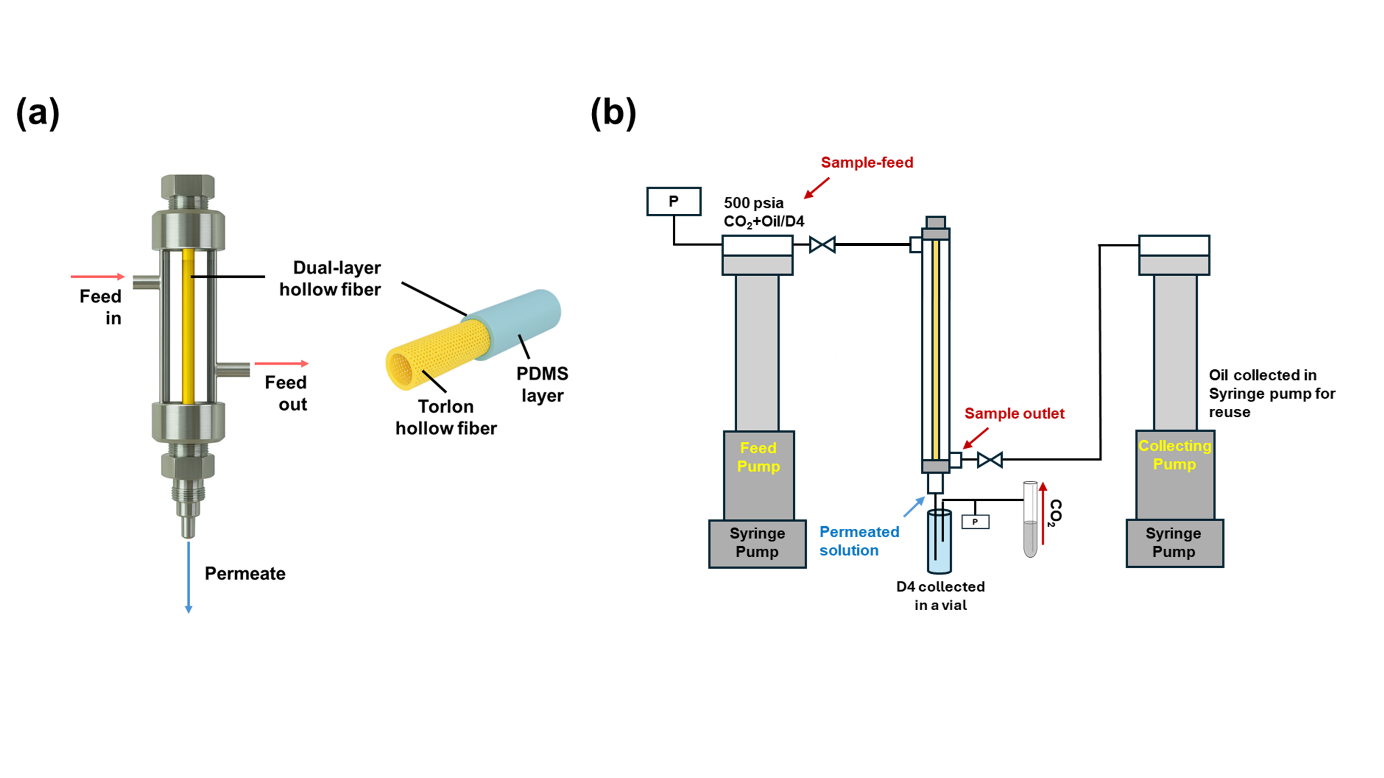


*Figure S1.* Sorp-vection system demonstration. (a) Schematic of the dual-layer hollow fiber module. (b) Schematic of the sorp-vection system.

Before sorp-vection process, the feed D4/silicone oil mixture is saturated with CO2 as described in Figure S2. The sorp-vection module features two distinct outlets. The first outlet allows the passage of the retentate solution through the shell side of the module where it then is sent to an accurate Flame Ionization Detector (FID) GC to analyze. The second outlet is specifically designed for the permeation of cyclic siloxanes and oil with CO_2_ that penetrate the PDMS layer as shown in Figure S1(a). The cyclic siloxanes can then be analyzed on a CO_2_-free basis. In an eventual version of this system the CO_2_ that permeates through the fiber can be captured in an additional step for compression and recycled to enable reuse and greatly reduce the CO_2_ requirements of the process. The CO_2_-diluted feed solution that passes through the module is collected in the collecting pump. The solution collected from the bore side will be monitored throughout the sorp-vection process. The recorded weight will be used to calculate the flux, and the permeate will subsequently be analyzed by GC to determine its composition.

Gas Chromatography Analysis of Sorp-vection Membrane Permeate

Following the completion of sorp-vection testing, the compositions of both retentate and permeate streams were characterized on a CO_2_-free basis using a GC (Agilent GC8860, USA), equipped with an FID. FID is particularly well-suited for detecting and quantifying hydrocarbons and other organic compounds, offering high sensitivity and reliability. To do the analysis, permeate and retentate samples were collected at designated intervals. These samples underwent a preparation process that included diluting high concentrations of D4 or oil to suitable levels in acetone. Once prepared, the samples were injected into the GC, where the concentrations of each component were quantitatively analyzed. The FID facilitated accurate detection of the separated components, providing detailed insights into the composition within the permeate and feed. This analysis accurately assessed the separation efficiency and selectivity of the dual-layer fibers, demonstrating their effectiveness in the sorp-vection process.


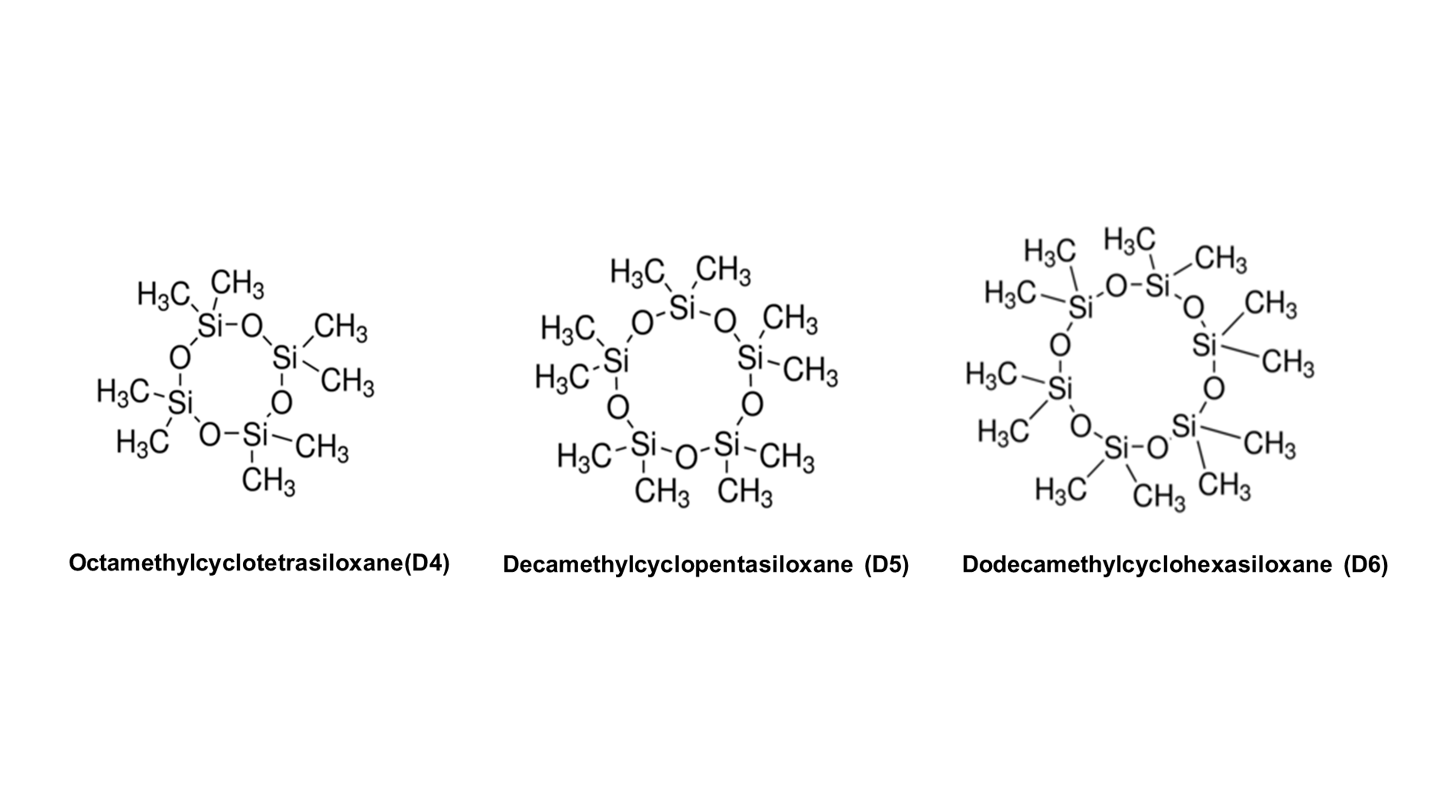


*Scheme S1.* Chemical structure of Octamethylcyclotetrasiloxane(D4), Decamethylcyclopentasiloxane (D5), Dodecamethylcyclohexasiloxane (D6)


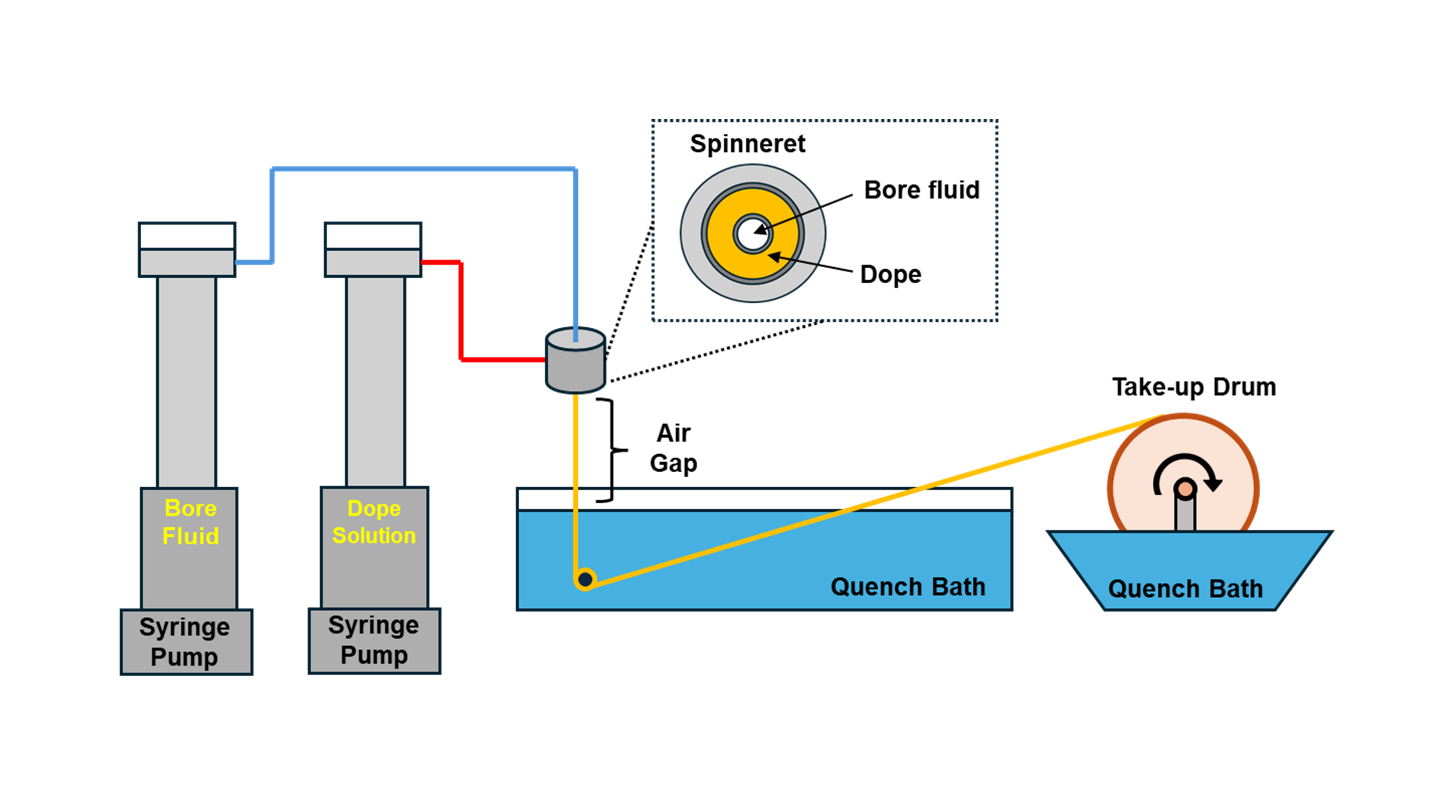


*Figure S2.* Fiber spinning system and procedures

During the spinning process, those solutions are coextruded from spinneret into an air gap(“dry-jet”) and immersed into an aqueous quench bath(“wet-quench”). The “dry-jet” step produces a dense skin layer while the “wet-quench” step forms the porous support structure. The outer diameter and inner diameter are determined by the spinning conditions, such as composition of bore fluid and dope solution, dope flow and bore flow of each solution into the spinneret and air gap height and take-up rate at the last stage on the take-up drum.


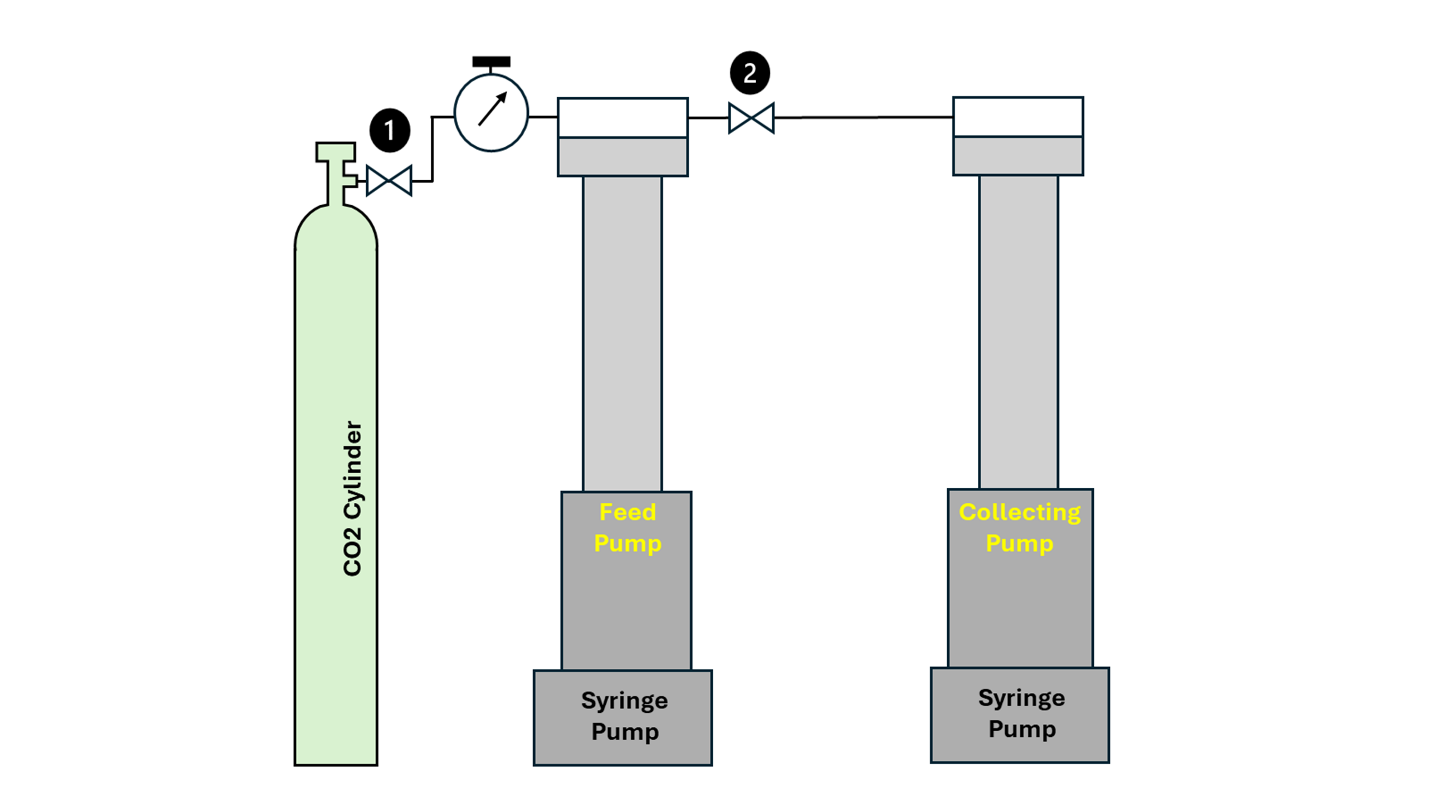


*Figure S3.* CO2 saturation process before sorp-vection process.

Prior to initiating the sorp-vection process, the D4/silicone oil mixture is first loaded into the feed pump. Valve 1 is then opened to introduce CO₂ into the feed solution. The system is pressurized to above 500 psia to ensure that sufficient CO₂ dissolves into the mixture, accounting for pressure reduction due to CO₂ diffusion during the process.

Then the Valve 2 is opened, the solution is continuously circulated through a fresh tubing loop connecting the feed pump and the collecting pump, providing adequate residence time for CO₂ saturation. After this circulation step, the D4/silicone oil mixture is fully saturated with CO₂ under high pressure and prepared for the sorp-vection process.


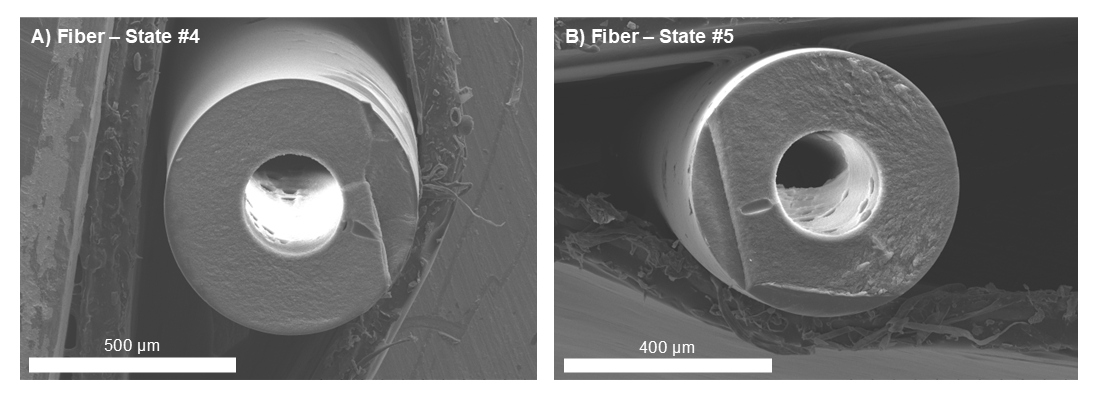


*Figure S4.* Torlon hollow fiber SEM image, A) State #4 fibers with an outer diameter of approximately 615 μm with a wall thickness of ~120 μm, B) State #5 fibers with outer diameter of ~500 μm and a thicker wall of ~150 μm.


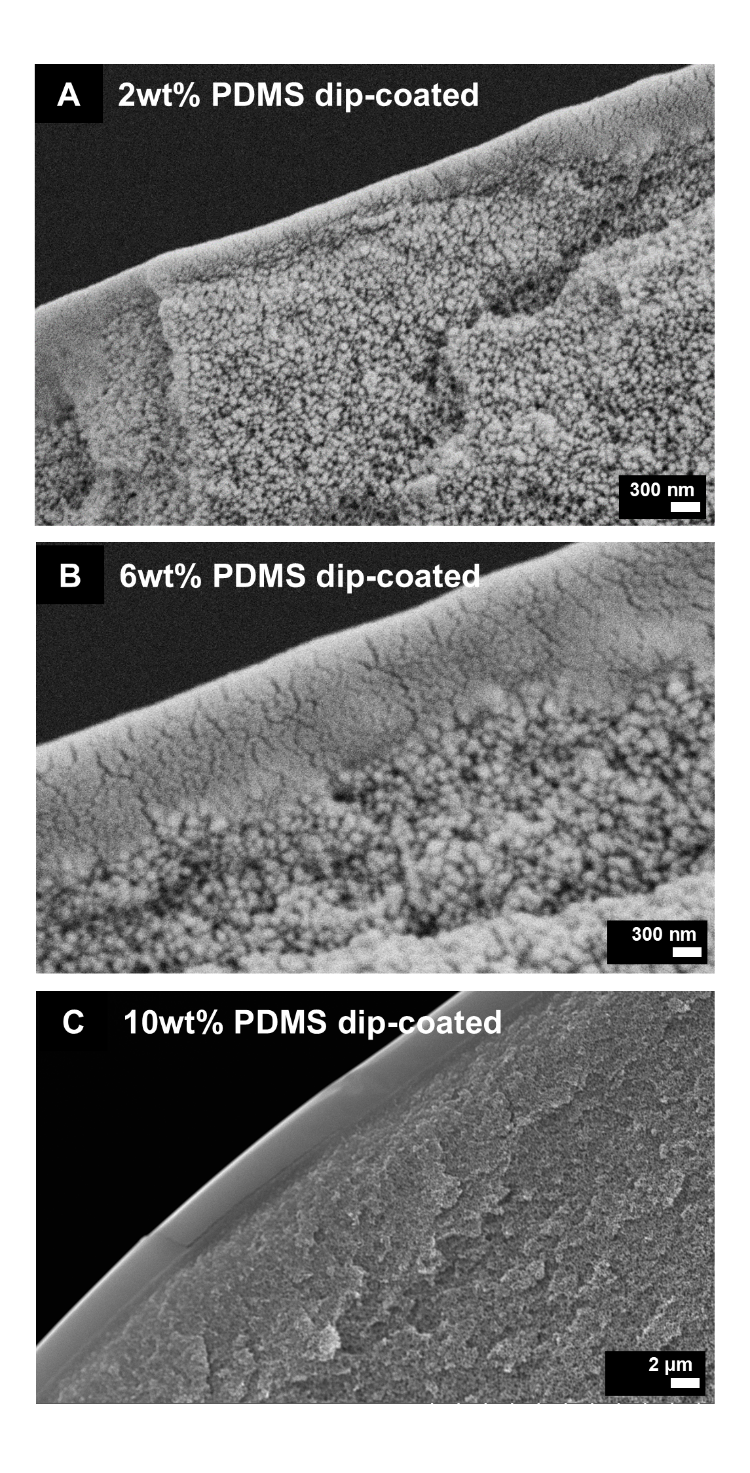


*Figure S5.* PDMS layer on the Torlon hollow fiber dip-coated with different concentration of PDMS in Heptane coated with solution of (A) 2wt% PDMS in Heptane, (B) 6wt% PDMS in Heptane, (C)10wt% PDMS in Heptane.


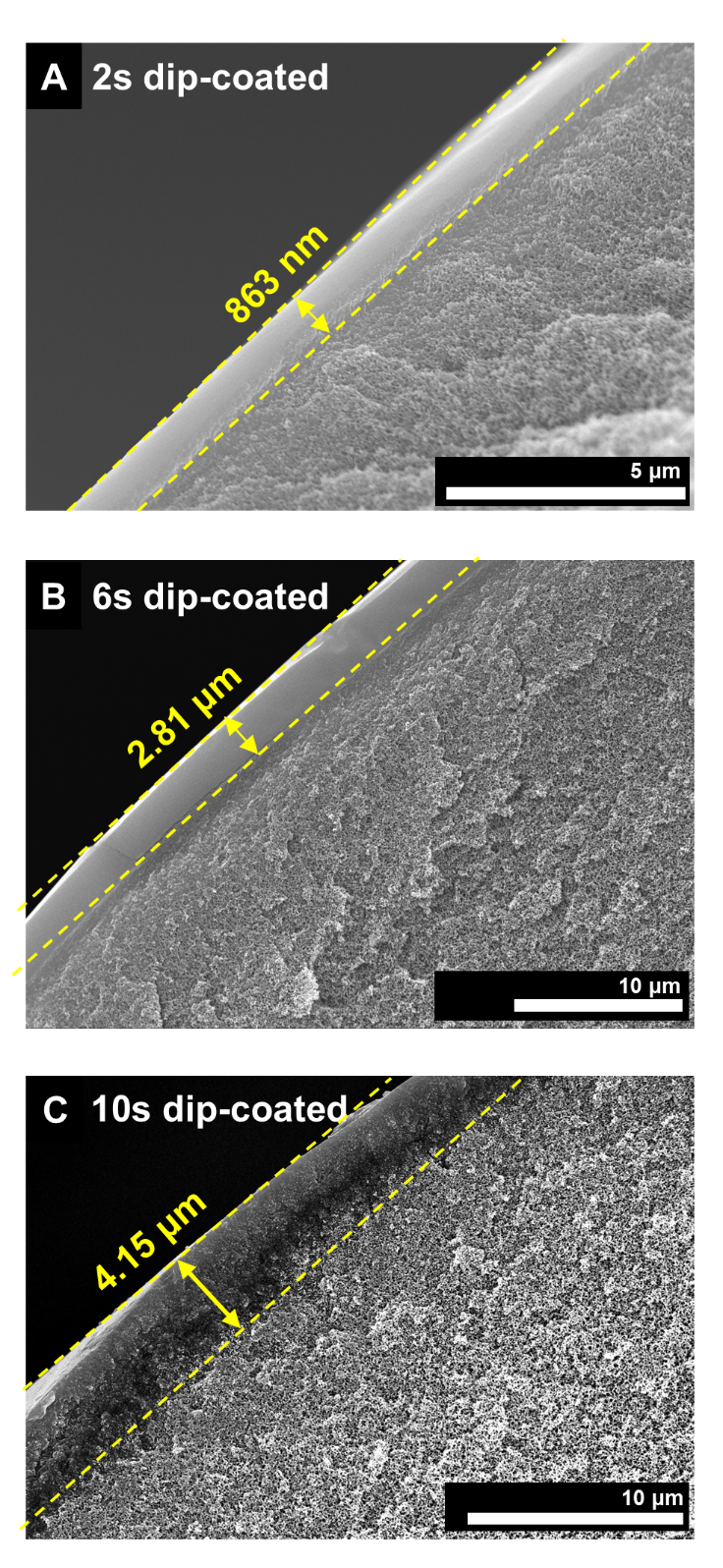


*Figure S6.* Thickness of PDMS layer on the Torlon hollow fiber with different dip-coating time in PDMS solution (A) 2 seconds dip-coated layer with thickness of 863nm, (B) 6 seconds dip-coated layer with thickness of 2.81μm, and (C) 10 seconds dip-coated layer with thickness of 4.15μm.


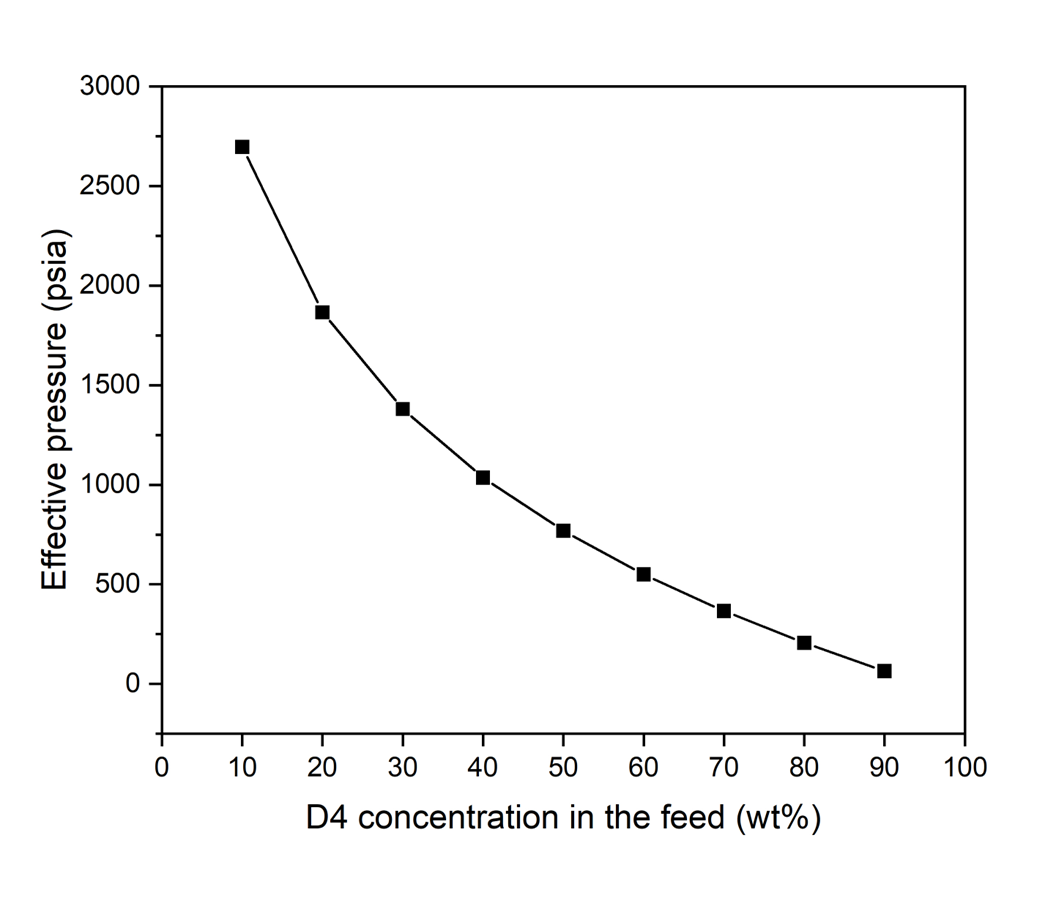


*Figure S7.* Minimum effective pressure required for separation, estimated from chemical potential differences based on D4 activity gradients across the membrane.


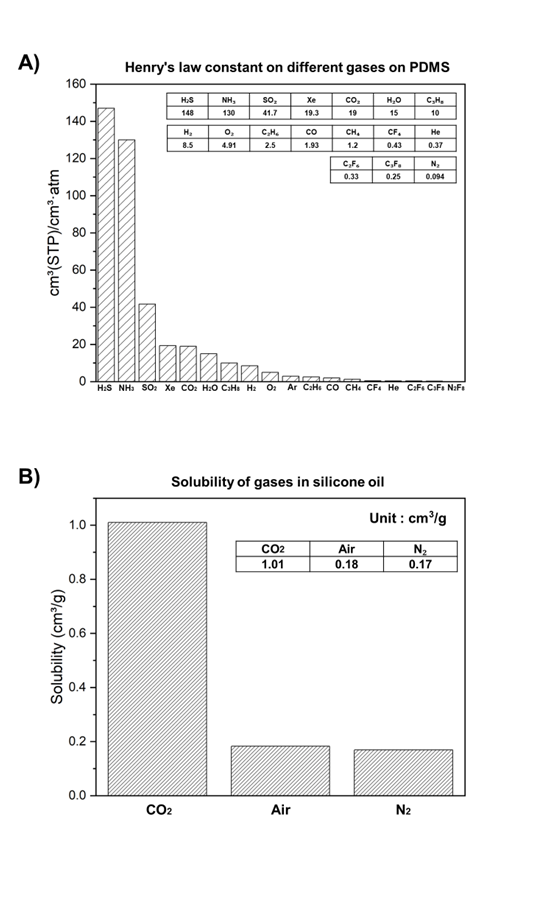


*Figure S8.* Solubility of different gases in PDMS and silicone oil ^[1,2]^

Selecting an appropriate sorp-vection agent is important to the sorp-vection process, as the driving force for sorp-vection is fundamentally influenced by two key factors: the gas’s permeability in PDMS and the transmembrane driving pressure difference. In addition, however, the sorp-vection agent permeability is also important when operated with the vacuum conditions, since this provides the in-situ flushing of permeates (oil and D4) out of the porous support.

Among various gases, CO₂ is particularly well-suited due to its high permeability in PDMS at 25°C.^[38]^ Although gases such as hydrogen sulfide (H₂S) and ammonia (NH₃) exhibit significant permeabilties in PDMS their use in practical sorp-vection experiments is not attractive due to safety and handling concerns. Considering both sorption performance and experimental feasibility, CO₂ represents the most reasonable and effective choice for driving the sorp-vection process. Moreover, the ability to recompress and recycle CO_2_ is appealing.

**Calculation of separation factors based on Sorp-vection theory.**

In 85wt% rich feed, the Equations (S3)-(S5) should be used to calculate the flux of D4 and high Mw silicone oil.

$$\begin{aligned} n_{A}=-\rho D_{A,m}\frac{d\omega_{A,m}}{dz}+\omega_{A,m}\left( n_{A}+n_{B}+n_{C} \right)\#\left( S3 \right) \end{aligned}$$

$$\begin{aligned} n_{B}=-\rho D_{B,m}\frac{d\omega_{B,m}}{dz}+\omega_{B,m}\left( n_{A}+n_{B}+n_{C} \right)\#\left( S4 \right) \end{aligned}$$

$$\begin{aligned} n_{C}=-\rho D_{C,m}\frac{d\omega_{C,m}}{dz}+\omega_{C,m}\left( n_{A}+n_{B}+n_{C} \right)\#\left( S5 \right) \end{aligned}$$

By using the liquid sorption data in the manuscript, we can know the mass of the membrane and mass of each component in the membrane. The weight fraction can be calculated as follows:

Here, we take

$$m_{CO2}=0.11g$$

$$m_{m}=1g$$

$$m_{D4,m}=1.61g$$

$$m_{Oil,m}=0.05g$$

$$m_{D4,Total}=2.61g$$

$$m_{Oil,Total}=1.05g$$

$$\omega_{CO2,m}=\frac{0.11}{1.11}=0.099$$

$\omega_{D4,m}=\frac{1.61}{2.61}*0.85=0.524$

$\omega_{Oil,m}=\frac{0.05}{1.05}*0.15=$0.00714

$$\omega_{m}=1-0.099-0.524-0.00714=0.370$$

*: Masses shown correspond to Fig. 5 with a 1gm PDMS polymer basis and the sorption isotherm reported for CO_2_ in PDMS in ref [37] in the manuscript.

Measured CO_2_ flux equals:

$n_{CO2}=\left( \frac{\mathbb{P}}{\mathcal{l}} \right)[\Delta p_{CO2}]=300\times{10}^{-6}\frac{486}{14.7}\left[ 76 \right]\left[ \frac{44}{22400} \right]=0.00148\frac{g}{{cm}^{2}s}$

By neglecting the diffusion term in $n_{A}$ and $n_{C}$, the equation (S3) and (S5) will be transformed as follows:

$$\begin{aligned} n_{Oil}=\omega_{Oil, m}\left( n_{Oil}+n_{D4}+0.00148g/{cm}^{2}s \right)\#\left( S6 \right) \end{aligned}$$

$$\begin{aligned} n_{D4}=\omega_{D4,m}\left( n_{Oil}+n_{D4}+0.00148g/{cm}^{2}s \right)\#\left( S7 \right) \end{aligned}$$

So, we will have 2 unknowns which are $n_{Oil}$ and $n_{D4}$. Then we can calculate those values and using $\omega_{Oil, m}$ and $\omega_{D4, m}$ in the equations. This gives $n_{Oil}=1.66*{10}^{-3}g/{cm}^{2}s$ and $n_{D4}=2.26*{10}^{-5}g/{cm}^{2}s$.

Then we can calculate the separation factor by using the Equation (9) in the manuscript,

$$\begin{aligned} S.F.=\frac{\left( \frac{n_{A}}{n_{C}} \right)_{Permeate}}{\left( \frac{\omega_{A}}{\omega_{C}} \right)_{Feed}}=\frac{1.66*{10}^{-3}/2.26*{10}^{-5}}{0.85/0.15}=12.96\#\left( S8 \right) \end{aligned}$$

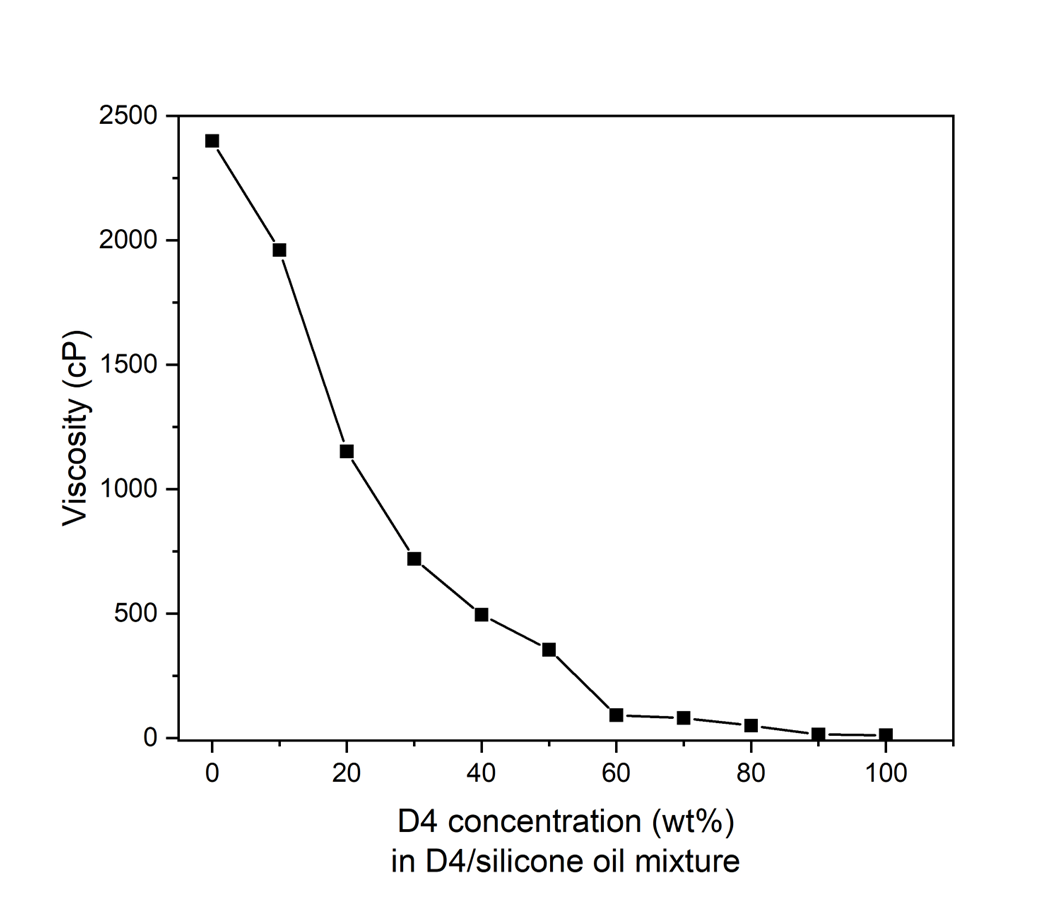


***Figure S9.*** Viscosity of D4 and silicone oil mixture according to the concentration of D4 in the mixture.

While the lower viscosity of 85 wt% D4 feed may partly facilitate permeation, we hypothesize that viscosity more directly influences internal concentration polarization (ICP). As oil content increases and viscosity rises, permeated oil accumulation within the porous support is expected to hinder CO_2_ transport, reducing effective permeate flux. Thus, viscosity may not only act as a bulk property influencing diffusion, but could also play a role in governing the extent of ICP in the sorp-vection system.

***Table S1.*** Fiber spinning conditions for state number #1 to #9.

| State # No. | Dope flow (mL/hr) | Bore flow (mL/hr) | Air gap (cm) | Take-up rate (m/min) |
| --- | --- | --- | --- | --- |
| 1 | 240 | 60 | 4 | 11 |
| 2 | 240 | 80 | 4 | 15 |
| 3 | 240 | 80 | 4 | 20 |
| 4 | 240 | 60 | 4 | 20 |
| 5 | 240 | 60 | 4 | 30 |
| 6 | 240 | 60 | 4 | 40 |
| 7 | 240 | 80 | 4 | 40 |
| 8 | 240 | 60 | 4 | 30 |
| 9 | 240 | 60 | 4 | 25 |

***Table S2.*** Pure gas permeation test on fiber state number #1 to #9.

| State # No. | OD (μm) | Permeance (He) (GPU) | Permeance (N_2_) (GPU) | Permeance (CO_2_) (GPU) | Selectivity (CO_2_/N_2_) |
| --- | --- | --- | --- | --- | --- |
| 1 | 753.9 | 8169.5 ± 38.4 | 4104.0 ± 17.0 | 3988.8 ± 12.1 | 0.97 |
| 2 | 614.0 | 3286.6 ± 3.1 | 1408.9 ± 3.6 | 1791.2 ± 17.5 | 1.3 |
| 3 | 544.0 | 1615.9 ± 2.8 | 636.0 ± 1.0 | 805.3 ± 0.2 | 1.3 |
| 4 | 618.1 | 538.0 ± 1.6 | 182.5 ± 0.2 | 313.0 ± 0.4 | 1.7 |
| 5 | 488.7 | 2253.5 ± 20.8 | 1211.6 ± 2.4 | 1474.4 ± 1.8 | 1.2 |
| 6 | 422.9 | 589.1 ± 0.8 | 226.3 ± 1.2 | 436.7 ± 0.5 | 1.9 |
| 7 | 397.8 | 5572.6 ± 13.0 | 4355.6 ± 4.5 | 4283.9 ± 14.6 | 1.0 |
| 8 | 420.8 | 1047.7 ± 1.9 | 404.5 ± 1.1 | 655.9 ± 1.2 | 1.6 |
| 9 | 405.1 | 771.1 ± 3.3 | 278.2 ± 1.7 | 505.5 ± 0.2 | 1.8 |

In Table S1, fibers from state #1 to #9 were fabricated with different conditions for fiber fabrication, and state #4 and state #5 fibers which showed the most uniform and stable structure were used in this study for dual-layer Torlon hollow fiber fabrications. Outer diameter and the permeance of fibers on He and N_2_, and selectivity for He/N_2_ were all checked as shown in Table S2 to characterize the defect-free nature of the PDMS layer.

***Table S3.*** Comparison of PDMS Molecular Weight: Pre-Cured vs. Non-Cured. (Error bars represent testing of different membrane modules (N=3))

|  | Mn (g/mol) | Mw (g/mol) | Mz (g/mol) | Mz+1 (g/mol) | Mw/Mn |
| --- | --- | --- | --- | --- | --- |
| 2% PDMS | 3,680 | 32,500 | 72,500 | 102,000 | 8.83 |
| 2% PDMS with  pre-curing | 4,660 | 36,200 | 75,100 | 108,000 | 7.77 |
| 6% PDMS | 4,730 | 59,000 | 144,000 | 228,000 | 12.47 |
| 6% PDMS with  pre-curing | 5,270 | 79,500 | 260,000 | 459,000 | 15.09 |
| 10% PDMS | 5,990 | 65,200 | 176,000 | 319,000 | 10.88 |
| 10% PDMS with  pre-curing | 4,650 | 105,000 | 499,000 | 938,000 | 22.58 |

***Table S4.*** Pure gas permeation test on PDMS coated fiber on fiber state #4-5. (Error bars represent testing of different membrane modules (N=3))

| State # No. | OD (μm) | Permeance (N_2_) (GPU) | Permeance (CO_2_) (GPU) | Selectivity (CO_2_/N_2_) |
| --- | --- | --- | --- | --- |
| 4 | 626.0 | 27.0 ± 0.5 | 243.1 ± 2.2 | 9.00 ± 0.03 |
| 5 | 438.6 | 14.6 ± 0.1 | 156 ± 0.7 | 10.70 ± 0.07 |

***Table S5.*** CO_2_-driven sorp-vection performance at different feed pressures.

| Pressure | Separation factor | Flux  (kg/m^2^hr) |
| --- | --- | --- |
| 100 psia | - | No measurable permeation |
| 200 psia | - | No measurable permeation |
| 300 psia | 6.67 ± 0.03 | 0.15 ± 0.01 |
| 400 psia | 14.25 ± 0.17 | 0.30 ± 0.12 |
| 500 psia | 13.36 ± 0.14 | 0.39 ± 0.19 |

***Reference :***

[1] W. Shi, N. S. Siefert, B. D. Morreale, *Journal of Physical Chemistry C* 2015, *119*, 19253–19265.

[2] L. Sprunger, A. Proctor, W. E. Acree, M. H. Abraham, *J Chromatogr A* 2007, *1175*, 162–173.
